# Low-Order Scaling Quasiparticle Self-Consistent GW for Molecules

**DOI:** 10.3389/fchem.2021.736591

**Published:** 2021-09-03

**Authors:** Arno Förster, Lucas Visscher

**Affiliations:** Theoretical Chemistry, Vrije Universiteit, Amsterdam, Netherlands

**Keywords:** GW approximation, convergence acceleration, analytical continuation, quasiparticle, quasiparticle self-consistent *GW*, DNA photodamage, theoretical spectroscopy

## Abstract

Low-order scaling GW implementations for molecules are usually restricted to approximations with diagonal self-energy. Here, we present an all-electron implementation of quasiparticle self-consistent GW for molecular systems. We use an efficient algorithm for the evaluation of the self-energy in imaginary time, from which a static non-local exchange-correlation potential is calculated via analytical continuation. By using a direct inversion of iterative subspace method, fast and stable convergence is achieved for almost all molecules in the GW100 database. Exceptions are systems which are associated with a breakdown of the single quasiparticle picture in the valence region. The implementation is proven to be starting point independent and good agreement of QP energies with other codes is observed. We demonstrate the computational efficiency of the new implementation by calculating the quasiparticle spectrum of a DNA oligomer with 1,220 electrons using a basis of 6,300 atomic orbitals in less than 4 days on a single compute node with 16 cores. We use then our implementation to study the dependence of quasiparticle energies of DNA oligomers consisting of adenine-thymine pairs on the oligomer size. The first ionization potential in vacuum decreases by nearly 1 electron volt and the electron affinity increases by 0.4 eV going from the smallest to the largest considered oligomer. This shows that the DNA environment stabilizes the hole/electron resulting from photoexcitation/photoattachment. Upon inclusion of the aqueous environment *via* a polarizable continuum model, the differences between the ionization potentials reduce to 130 meV, demonstrating that the solvent effectively compensates for the stabilizing effect of the DNA environment. The electron affinities of the different oligomers are almost identical in the aqueous environment.

## 1 Introduction

The *GW* approximation (GWA) to Hedin’s equations (Hedin, 1965) is a popular approach to calculate charged excitations in molecular systems. Recent applications include the calculation of band gaps and elucidation of charge-transfer in organic donor-acceptor compounds (Blase and Attaccalite, 2011; Blase et al., 2011; Caruso et al., 2014), applications to dye-sensitized solar cells (Marom et al., 2011; Faber et al., 2012; Umari et al., 2013; Marom et al., 2014; Mowbray and Migani, 2015), electronic level alignment in photocatalytic interfaces (Migani et al., 2013, 2014), core-ionization spectra of medium sized molecules (Van Setten et al., 2018; Golze et al., 2018, 2020) or photo-electron spectra of transition metal oxides (Berardo et al., 2017; Hung et al., 2017; Shi et al., 2018; Rezaei and Ögüt, 2021). Combined with the Bethe-Salpeter equation (BSE) formalism (Salpeter and Bethe, 1951; Strinati, 1988) the GWA has been used to calculate optical spectra of Cyanins (Boulanger et al., 2014), the Bacteriochlorin molecule (Duchemin et al., 2012) or Bacteriochlorophylls and Chlorophylls (Hashemi and Leppert, 2021). At the same time, the GWA has been implemented into an increasing number of molecular electronic structure codes (Ke, 2011; Caruso et al., 2012; Caruso et al., 2013; Ren et al., 2012; Van Setten et al., 2013; Kaplan et al., 2015, 2016; Bruneval et al., 2016; Wilhelm et al., 2016; Tirimbò et al., 2020b). Traditionally, these implementations use localized basis functions and the resolution-of the identity or density fitting approximation (Baerends et al., 1973; Whitten, 1973; Dunlap et al., 1979) within the global Coulomb metric (RI-V) (Vahtras et al., 1993), leading to a scaling of *N* ^4^ with system size. Systems of around 100 atoms are within reach on standard hardware (Knight et al., 2016), while highly parallel implementations enable applications to systems with more than 300 atoms on modern supercomputers (Wilhelm et al., 2016; Wilhelm et al., 2018; Wilhelm et al., 2021).

Over the last years, many algorithms with reduced asymptotic scaling with system size have been proposed. These are usually based on the space-time approach by Godby and coworkers (H. N. Rojas et al., 1995; Rieger et al., 1999). The original space-time method is based on the observation that it is much simpler to solve the Dyson equations in the GWA in reciprocal space and imaginary frequency while the kernels of these Dyson equations are most easily evaluated in real space and imaginary time, reducing the asymptotic scaling of the GWA to *N* ^3^. Building on earlier work by Almlöf (Almlöf et al., 1982), Kresse, Kaltak and coworkers could significantly reduce the prefactor of these calculations by using non-uniform spaced grids in imaginary time and imaginary frequency and an efficient way to switch between both domains (Kaltak et al., 2014a; Kaltak et al., 2014b; Kaltak and Kresse, 2020). Over the last years, there has been a surge of new *GW* implementations based on the space-time method for periodic (Kutepov et al., 2012; Chu et al., 2016; Liu et al., 2016; Kutepov et al., 2017; Grumet et al., 2018; Kutepov, 2020; Singh and Wang, 2020; Foerster and Gueddida, 2021) and finite (Wilhelm et al., 2018; Koval et al., 2019; Förster and Visscher, 2020; Duchemin and Blase, 2021; Wilhelm et al., 2021) systems. Other recent examples of low-order scaling implementations include the spectral function based approach by Foerster et al. (2011), the time-shredded propagator formalism by Ismail-Beigi and coworkers (Kim et al., 2020), stochastic *GW* developed by Neuhauser et al. (2014), Vlček et al. (2017), Vlček et al. (2018), Weng and Vlcek (2021), and also a fragment molecular orbital based implementation (Fujita et al., 2019).

For molecular systems, diagonal approximations to the self-energy are commonly made. They rely on the assumption that the wave function of generalized Kohn-Sham (KS) density functional theory (DFT) is similar to the GW wave function. One then evaluates corrections to the DFT single orbital energies by calculating the diagonal elements of the self-energy matrix Σ. The most economical way to calculate these corrections is the one-shot *G*_0_ *W*_0_ approach which heavily depends on the mean-field starting point. Extensive benchmarks (Marom et al., 2012; Bruneval and Marques, 2013; Caruso et al., 2016; Knight et al., 2016) have provided substantial evidence that hybrid functionals with a rather large amount of exact exchange or long-range corrected hybrids are usually a suitable starting point. In addition, non-empirical procedures to select an optimal starting point for a given system have been proposed (Gallandi and Körzdörfer, 2015; Dauth et al., 2016; Bois and Körzdörfer, 2017). Finally, in eigenvalue-only self-consistent *GW* (evGW) the QP energies are updated until they are stationary, removing the starting point dependence to a large extent.

QP energies calculated following these strategies are almost always more accurate than fully self-consistent *GW* (scGW) calculations for molecules. As discussed by Kotani, van Schilfgaarde and Valeev, QP approximations, i.e. approximations in which satellites are neglected, emphasize the importance of the Ward identity (Ward, 1950) in the long-range and low-frequency limit. The Ward identity demands ’*Z*-factor cancellation’ (Kotani et al., 2007) between the three-point Vertex and the renormalized electron propagator. *Z* is the QP renormalization factor. In QP approximations, neither the vertex is included nor is the propagator renormalized, and the effect of both approximations cancel in the above-mentioned limit. This limit can be expected to be of particular importance for weakly correlated molecules to which the GWA is frequently applied.

As opposed to diagonal approximations, scGW is strictly starting point independent and also allows to calculate 1-particle reduced density matrices (1RDM) including electron correlation effects from first principles. Most importantly, it does not contain any adjustable parameters. Another method which also offers these advantages is the QP self-consistent *GW* (qs*GW*) method by Kotani, van Schilfgaarde and Faleev. (Van Schilfgaarde et al., 2006; Kotani et al., 2007). qs*GW* can be seen as a non-empirical procedure to find an optimal starting point for a *G*_0_ *W*_0_ calculation. This is accomplished by mapping the *GW* self-energy self-consistently to a non-local, Hermitian, and static exchange-correlation potential. This potential has been shown to be optimal in a variational sense (Ismail-Beigi, 2017). Diagonalization of the resulting mean-field Hamiltonian yields eigenvectors and eigenvalues from which a new non-interacting Green’s function is obtained. This self consistent field (SCF) procedure is reminiscent of generalized KS theory, with the notable difference that the exchange-correlation potential is not a functional of the 1RDM but rather of the non-interacting single-particle Green’s function. qs*GW* is starting point independent and fulfills the Ward identity in the low frequency and long range limit.

In canonical implementations (Ke, 2011; Bruneval, 2012; Koval et al., 2014; Kaplan et al., 2015; Kaplan et al., 2016), the need to calculate the off-diagonal elements of the self-energy matrix and the fact that it is typically more difficult to converge make qs*GW* typically an order of magnitude more expensive than ev*GW* (Gui et al., 2018) which in turn is typically 5–10 times more expensive than *G*_0_ *W*_0_ due to the requirement of self-consistency. Moreover, low-order scaling implementations for molecules are typically restricted to diagonal approximations only (Wilhelm et al., 2018; Förster and Visscher, 2020; Duchemin and Blase, 2021; Wilhelm et al., 2021) To fill this gap, we extend the recently developed low-order scaling diagonal *GW* implementation in ADF (Baerends et al., 2020; Förster and Visscher, 2020, Förster and Visscher, 2021) to qs*GW*. We evaluate the qs*GW* self-energy as a direct product in imaginary time, in the same way as in the diagonal approximation. Even though the qs*GW* self-energy is static, for larger systems evaluation of the self-energy at an array of imaginary time points is more efficient than its evaluation at a single real frequency point. The procedure is similar to the linearized qs*GW* method by Kutepov and coworkers (Kutepov et al., 2017) which is also based on the imaginary time formalism and in which the self-energy is averaged over all frequencies. However, in our implementation, we only average over frequencies for the off-diagonal elements but retain the optimum exchange-correlation potential on the diagonal. We achieve stable and rapid convergence of the SCF procedure by a suitable implementation of the direct inversion in the iterative subspace (DIIS) Pulay (1980) approach. Most importantly, the proposed algorithm is easy to implement and only requires to combine the qs*GW* approach with the space-time implementation for the self-energy and an efficient method to evaluate the exact exchange-contribution to the Fock matrix.

This work is organized as follows: In section 2 we first recapitulate the qs*GW* procedure and describe some aspects of our implementation. We focus on the implementation of the DIIS and on the analytical continuation (AC) of the self-energy. In section 3, we confirm the correctness of our implementation by comparison to ionization potentials (IP) (Kaplan et al., 2016) from TURBOMOLE (Balasubramani et al., 2020) and investigate the convergence of the SCF equations. We also illustrate the computational performance of our implementation with a proof-of principle application to large DNA oligomers. In section 4 we summarize and conclude this work.

## 2 Methods

In this section, we review the qs*GW* method and comment on our implementation, focusing on the AC of the self-energy as well as our approach to accelerate convergence of the SCF procedure. Greek lowercase letters *μ*, *ν* … label atomic orbitals (AO) and run from 1 to *n*_*AO*_. Latin lowercase letters *p*, *q*, *r*, … label general MOs and run from 1 to *n*_*MO*_. *i*, *j*, *k* (*a*, *b*, *c*) label occupied (virtual) MOs and run from 1 to *N*_*occ*_ (*N*_*virt*_). Latin symbols without labels denote tensors in some basis which will always be clear from the context.

### 2.1 QP Self-Consistent GW

The GWA is an approximation to the self-energy appearing in Dyson’s equation (Dyson, 1949),

$$\underset{r}{\sum }\Sigma_{pr}\left(\omega_{p}\right)U_{rq}\left(\omega_{p}\right)=\left[\omega_{p}-\epsilon_{p}\right]U_{pq}\left(\omega_{p}\right).$$ (1)

We mostly work in a basis of molecular orbitals (MO),

$$\phi_{p}^{\left(n\right)}\left(r\right)=\underset{\mu }{\sum }\chi_{\mu }\left(r\right)b_{\mu p}^{\left(n\right)},$$ (2)

where the *χ*_*μ*_ are AOs. Dysons’s equation is non-linear and will be solved *via* a fixed point iteration. The superscript (*n*) means that we are in the *n*th iteration of a SCF procedure. The self-energy Σ is non-Hermitian and energy dependent. Thus, *U* is complex and energy dependent as well. We will neglect spin in the following.

The *ε*_*p*_ are obtained from solving the generalized KS problem,

$$\underset{\nu }{\sum }H_{\mu \nu }^{\left(0\right)}b_{\nu p}^{\left(0\right)}=\underset{\nu }{\sum }S_{\mu \nu }b_{\nu p}^{\left(0\right)}\epsilon_{p}^{\left(0\right)}\;,\;H_{KS}=T+V_{ext}+V_{Hxc}\left[P\right],$$ (3)

where *V*_*Hxc*_ is the sum of exchange-correlation potential *V*_*xc*_ and Hartree potential *V*_*H*_, being functionals of the 1RDM *P* and the electron density, respectively. *T* and *V*_*ext*_ are kinetic energy and external potential, respectively. *S* is the overlap matrix of AOs and *b* defines a transformation from AO to MO basis,

$$M_{pq}=b_{p\mu }M_{\mu \nu }{\left[b^{†}\right]}_{\nu q}.$$ (4)

In the AO basis, *P* is given as

$$P_{\mu \nu }=2\overset{N_{occ}}{\underset{i}{\sum }}b_{\mu i}{\left[b^{†}\right]}_{i\nu }.$$ (5)

We also define the Hamiltonian of the Hartree approximation,

$$H_{H}=H_{KS}-V_{xc}.$$ (6)

The Green’s function *G*_0_ corresponding to the non-interacting Hamiltonian is diagonal in the MO basis with

$${\left[G_{0}\right]}_{pp}\left(\omega \right)={\left[i\omega -\epsilon_{p}\right]}^{-1}.$$ (7)

We can then expand Σ in terms of *G*_0_ as follows (Hedin, 1965; Martin et al., 2016),

$$\Sigma \left(\omega \right)=\left(G_{0}*W_{0}\right)\left(\omega \right)+\ldots ,$$ (8)

and in the GWA the expansion is truncated after first order. *W*_0_ is the screened Coulomb interaction, calculated in the bubble approximation (Onida et al., 2002) from *G*_0_ (Hedin, 1965). Without further approximations to Σ, one typically avoids solving (Eq. 1) but instead calculates the interacting Green’s function *G* by inversion of

$${\left[G\left(\omega \right)\right]}^{-1}={\left[G_{0}\left(\omega \right)\right]}^{-1}-\Sigma \left(\omega \right).$$ (9)

From there one proceeds by building the self-energy (Eq. 8) but replaces *G*_0_ by *G*, and *W*_0_ by *W* and repeats this procedure until self-consistency is reached. In more approximate GW schemes, one avoids solving (Eq. 9). In diagonal approximations to Dysons’s equation, one assumes Σ to be diagonal. In that case, *U* in (Eq. 1) is unity for all *ω* and (Eq. 1) reduces to a set of independent non-linear equations for *ω*. In qsGW on the other hand, one does not make the diagonal approximation but Σ is mapped to a Hermitian and frequency-independent exchange-correlation potential $V_{xc}^{qsGW}$. For this mapping, it is convenient to define

$$W_{0}\left(\omega \right)=V_{c}+{\tilde{W}}_{0}\left(\omega \right),$$ (10)

with *V*_*c*_ being the bare Coulomb potential. The self-energy can then be decomposed into a static and dynamic part

$$\Sigma \left(\omega \right)=\Sigma_{x}+\left(G_{0}*{\tilde{W}}_{0}\right)\left(\omega \right)=\Sigma_{x}+\Sigma_{c}\left(\omega \right).$$ (11)

Σ_*x*_ is the Fock exchange potential, $V_{x}^{qsGW}=\Sigma_{x}$, and following Kotani et al. (2007), the correlation part of *V*_*xc*_ is obtained from Σ by taking one of the real symmetric definitions

$${\left[V_{c}^{qsGW}\right]}_{pq}=\frac{1}{2}\left[Re\;{\left[\Sigma_{c}\right]}_{pq}\left(\epsilon_{p}\right)+Re\;{\left[\Sigma_{c}\right]}_{pq}\left(\epsilon_{q}\right)\right],$$ (12)

or

$${\left[V_{c}^{qsGW}\right]}_{pq}=\delta_{pq}Re\;{\left[\Sigma_{c}\right]}_{pq}\left(\epsilon_{p}\right)+\left(1-\delta_{pq}\right)Re\;{\left[\Sigma_{c}\right]}_{pq}\left(\omega =0\right).$$ (13)

There are formal reasons why (Eq. 12) should be preferred over (Eq. 13). Constructing the qs*GW* Hamiltonian *via* (Eq. 12) minimizes the length of the gradient of the Klein functional (Klein, 1961) with respect to *G*_0_ (Ismail-Beigi, 2017) and can be seen as an optimized effective non-local potential. The approach bears strong resemblance to what is usually referred to as the optimized effective potential (OEP) method (Talman and Shadwick, 1976). Another possibility is to linearize the self-energy around the chemical potential. This has been implemented by Kutepov et al. (2017). Physically, it is equivalent to taking the static limit of the self-energy, or averaging over frequencies. We will discuss in more detail below that such an approach has advantages with regards to numerical stability. However, we think that one should use the optimum potential at least for the diagonal elements. (Eq. 13) is a hybrid between (Eq. 12) and Σ (*ω* = 0) which retains the optimum potential on the diagonal. Employing (Eq. 13) can be justified if one assumes that the effect of using the optimum potential as opposed to Σ (*ω* = 0) will cancel out to a large extent for the off-diagonal elements. We provide numerical evidence later on that this is indeed true. Also an approach using Löwdin’s orthogonalization has been proposed to construct the QP Hamiltonian (Sakuma et al., 2009) but that construction is not considered here.

With these simplifications, we can now solve (Eq. 1) self-consistently. In each iteration, we solve

$$\underset{r}{\sum }H_{pr}^{qsGW^{\left(n+1\right)}}U_{rq}^{\left(n+1\right)}=\omega_{p}^{\left(n+1\right)}U_{pq}^{\left(n+1\right)},$$ (14)

with

$$H^{qsGW^{\left(n+1\right)}}=H_{H}+\Delta V_{H}^{\left(n+1\right)}+V_{xc}^{qsGW^{\left(n+1\right)}}$$ (15)

and

$$V_{xc}^{qsGW^{\left(n+1\right)}}=V_{x}\left[P^{\left(n\right)}\right]+V_{c}^{qsGW}\left[G_{0}^{\left(n\right)}\right].$$ (16)

In each iteration, *H* ^*qsGW*^ is expressed in the basis in which $G_{0}^{\left(n\right)}$ is diagonal. That is, at the *n* + 1st iteration, *H* ^*qsGW*^ is expressed in terms of the $\left\{\phi_{i}^{\left(n\right)}\right\}$ and unless self-consistency has been reached, *U* ^(*n*)^ will not be unity and defines a rotation of the molecular orbitals. We now set

$$\begin{array}{cc} b_{\mu p}^{\left(n+1\right)}= & \underset{q}{\sum }b_{\mu q}^{\left(n\right)}U_{qp}^{\left(n+1\right)}\\ \epsilon_{p}^{\left(n+1\right)}= & \omega_{p}^{\left(n+1\right)}\;\forall p \end{array}$$ (17)

and evaluate $G_{0}^{\left(n+1\right)}$ *via* (Eq. 7) which in turn is used to evaluate (Eq. 11) and finally (Eq. 12) or (Eq. 13). *P* ^(*n*+1)^ is then evaluated from (Eq. 5) and the change in the Hartree-potential is calculated as

$$\Delta V_{H}^{\left(n+1\right)}=V_{H}\left[\Delta P^{\left(n+1\right)}\right],$$ (18)

with

$$\Delta P^{\left(n+1\right)}=P^{\left(n+1\right)}-P^{\left(n\right)}.$$ (19)

The cycle is repeated until self-consistency is reached.

### 2.2 Implementation

As already stressed in the introduction, for the qsGW implementation no modifications of the code described in Förster and Visscher (2020) for the calculation of the self-energy are needed. A description of the algorithm can be found in Förster and Visscher (2020) and in Förster and Visscher (2021) we reported important modification of our original implementation, increasing accuracy and robustness. The only points we discuss hered are related to the convergence and stability of the self-consistent field (SCF) procedure.

#### 2.2.1 Analytical Continuation

In space-time implementations of the GWA, the self-energy is evaluated in imaginary time and then Fourier transformed to the imaginary frequency axis. In ADF, the self-energy is calculated in the AO basis on a non-uniform grid of imaginary time points. After transformation to the reference basis [the MO basis from the generalized KS calculation in the first iteration and the basis defined by (Eq. 17) later], the self-energy matrix is Fourier transformed to a non-uniform grid in imaginary frequency space. For the implementation of this transformation, we refer to Kaltak et al. (2014b) and to the appendix of Förster and Visscher (2021). Since the non-uniform grids depend on the QP energies used to build *G*_0_ we also need to recalculate these grids at the beginning of each qs*GW* iteration to ensure independence of the results from the initial guess.

After this transformation, Σ is known on a discrete set of points $W={\left\{i\omega_{\beta }\right\}}_{\beta =1,N_{\omega }}$ on the imaginary frequency axis. However, to evaluate Eq. 13, we need to know the self-energy on the real frequency axis at the positions of the QP energies $\epsilon_{p}^{\left(n\right)}$. To this end, we seek to find a function *f* which is analytic in the largest possible domain A⊂C and coincides with Σ in W. For a meromorphic function (as the self-energy) which is known on the whole imaginary axis, it is always possible to find such a function so that A=C, but since we only know the self-energy on a small subset of points, only an approximate solution can be found. The problem here is, that the AC is exceptionally ill-conditioned, i.e. numerical noise in the input data might significantly affect the output (Shinaoka et al., 2017).

Among the many developed algorithms [see for instance Levy et al. (2017) for an overview], the construction of a continued fraction (Vidberg and Serene, 1977; Beach et al., 2000) via a Padé approximant is most common in implementations of the GWA. While in many codes Thiele’s reciprocal difference method is implemented, (Liu et al., 2016; Grumet et al., 2018; Foerster and Gueddida, 2021), ADF, implements the variant by Vidberg and Serene (Vidberg and Serene, 1977), which for example has also been implemented by Kutepov (Kutepov, 2020). In the latter variant, the coefficients of the continued fraction are calculated while the former method returns the value of the continued fraction (Beach et al., 2000). While it has been claimed that the former variant is numerically more stable (Liu et al., 2016), we did not experience any numerical issues with our implementation for diagonal self-energies. This procedure typically yields good results for states close to the HOMO-LUMO gap while it becomes unreliable for core states (Golze et al., 2018, 2020). Exceptions are cases for which the self-energy has a pole close to the position of the QP energy (Govoni and Galli, 2018). Partial self-consistency in *G* pushes the poles away from the QP peak (Golze et al., 2019), and consequently, these issues should not be present in qs*GW* as well. This is different from situations in which the independent QP picture breaks down and the spectral weight of a single excited electrons is distributed between multiple peaks. The former is a purely numerical issue while the latter is caused by strong correlation and can not be overcome by partial self-consistency. It has also been shown in Wilhelm et al., 2021 that AC yields accurate results for semi-core and inner valence states in case the real part of the self-energy does not have poles in the vicinity of the QP solutions.

If one is only interested in accurate valence states, AC *via* Padé approximants is not problematic for *G*_0_ *W*_0_ where (Eq. 1) reduces to a set of *N* independent non-linear equations where *N* is the number of MOs. In ev*GW*, the situation is only slightly different. The *N* equations are still independent, but information from all QP energies enters the polarizability so that there is an implicit dependence of the QP energies on each other. In practice, this is also not an issue since the numerical errors are typically orders of magnitude smaller than the absolute values of the QP energies.

The situation is different for qs*GW*. The Off-diagonal elements of Σ_*c*_ are often equal to or very close to zero (Kaplan et al., 2015) and generally small compared to the diagonal elements. For these off-diagonal elements, numerical errors from AC can be orders of magnitudes larger than the values of the off-diagonal elements. Since there are many of them, this might significantly alter the solutions of Eq. 14. Due to the non-linear nature of the QP equations, this can complicate convergence of the SCF procedure or even lead to erroneous results. The development of more reliable methods for AC is a very active field of research (Bergeron and Tremblay, 2016; Levy et al., 2017; Otsuki et al., 2017; Gull et al., 2018; Fournier et al., 2020; Fei et al., 2021) and it would certainly be interesting to investigate whether other techniques are more suitable for qsGW. For now, we restrict ourselves to the techniques of Padé-approximants. To ensure numerical stability, two aspects need to be considered:

First, it seems reasonable to assume that AC close to the Fermi energy is also more reliable for the off-diagonal elements of Σ. To this end, using (Eq. 13) to construct the exchange-correlation potential seems to be more suitable for our implementation than (Eq. 12). As we will see later on, both constructions of the exchange-correlation potential lead to similar results, but using (Eq. 13), the SCF procedure is significantly easier to converge. In fact, applying the same reasoning one could justify to use Σ (*ω* = 0) (Kutepov et al., 2017) instead. However, as we will show below, using (Eq. 13) is sufficiently numerically stable.

Second, after evaluating Eq. 13 or (Eq. 12), numerical noise needs to be removed rigorously from $V_{c}^{qsGW}$. At self-consistency, the off-diagonal elements of $V_{c}^{qsGW}$ need to be zero: In the *n* + 1 the iteration, $V_{c}^{qsGW}$ is expressed in the basis which diagonalizes the operator defined in (Eq. 15) in the *n*th iteration. At self-consistency *b* ^(*n*+1)^ = *b* ^(*n*)^, which will not be the case when the off-diagonal elements of $V_{c}^{qsGW}$ will be different from zero. In our present implementation, we set all values with magnitude smaller than 1*e* ^−6^ to zero. This cut-off is of the order of the numerical noise introduced by the AC. As we will show later on, despite this drastic cut-off the HOMO and LUMO energies can be converged to a degree that the QP energies are converged within a few meV.

#### 2.2.2 Convergence Acceleration

As outlined so far, in each iteration of the self-consistency cycle the previous qs*GW* Hamiltonian is replaced by the new one, similar to the Roothaan algorithm for the Hartree-Fock (HF) equations. For Hartree-Fock, it is well known, that such a procedure can be numerically unstable (Cances and Le Bris, 2000) and convergence difficulties are encountered already for the simplest molecules (Koutecký and Bonačić, 1971; Bonačić-Koutecký and Koutecký, 1975). Also in many *GW* implementations, convergence has been shown to be much slower than with a simple linear mixing scheme (Caruso et al., 2013; Kaplan et al., 2016). While the latter seems to work reasonably well for ev*GW* (Gui et al., 2018), it seems that there is room for improvement for qs*GW* (Gui et al., 2018). An iterative fixed point procedure of the general form

$${\left\{G_{0}^{\left(m\right)}\right\}}_{0\leq m\leq n+1}\to {\tilde{H}}^{qsGW^{n+1}}\to \epsilon^{\left(n+1\right)}\;,b^{\left(n+1\right)}$$ (20)

is clearly a better option. A practical way to implement this is to replace (Eq. 14) by

$$\underset{r}{\sum }{\tilde{H}}_{pr}^{qsGW^{\left(n+1\right)}}U_{rq}^{\left(n+1\right)}=\omega_{p}^{\left(n+1\right)}U_{pq}^{\left(n+1\right)},$$ (21)

with

$${\tilde{H}}^{qsGW^{\left(n+1\right)}}=\overset{n+1}{\underset{m=n-n_{0}}{\sum }}\alpha_{m}H^{qsGW^{\left(m\right)}},$$ (22)

where

$$\overset{n}{\underset{m=n-n_{0}}{\sum }}\alpha_{m}=1,$$ (23)

needs to be fulfilled and *n*_0_ is the maximum number of previous iterations taken into account. We determine the expansion coefficients *α*_*m*_ using Pulay’s DIIS method (Pulay, 1980). In the DIIS method, we seek to minimise the residual error

$$r^{\left(n+1\right)}=\overset{n}{\underset{m=n-n_{0}}{\sum }}\alpha_{m}r^{\left(m\right)},$$ (24)

subject to the constraint Eq. 23. One might additionally require the *α*_*m*_ to be positive (what is usually called EDIIS) (Kudin et al., 2002) but we did not find any improvement over the simple DIIS. Different implementations of DIIS differ in the definition of the residual error. Since *G*_0_ uniquely determines *H* ^*qsGW*^, we would ideally define

$$r^{\left(n+1\right)}=G_{0}^{\left(n+1\right)}-G_{0}^{\left(n\right)},$$ (25)

however, storage (or recalculation) of this quantity for *n*_0_ iterations is inefficient. Therefore, one can use

$$r^{\left(n+1\right)}=P^{\left(n+1\right)}-P^{\left(n\right)},$$ (26)

which is related to the time-ordered Green’s function by taking the limit *τ* → 0^−^ (*τ* is the difference between both time arguments). In this work, we have used a different definition for the residual which is, however, identical to (Eq. 26).^1^

Technically, in the *n*th iteration we solve (Eq. 14) and evaluate the corresponding *b* ^(*n*)^ from which we calculate *P* ^(*n*)^ and *Q* ^(*n*)^. We check for convergence by evaluating the Frobenius norm of the residual (Eq. 26),

$$N_{F}=\frac{1}{N_{MO}^{2}}\sqrt{\underset{\mu \nu }{\sum }{\left[r_{\mu \nu }^{\left(n+1\right)}\right]}^{2}},$$ (27)

and terminate the SCF as soon as *N*_*F*_ < *ε*_*SCF*_ for two subsequent iterations. As we will show later on, *ε*_*SCF*_ = 1*e* ^−7^ leads to QP energies which are converged within a few meV for all systems in the GW100 database (Van Setten et al., 2015). Subsequently, we store *r* ^(*n*+1)^ and $H^{qsGW^{\left(n+1\right)}}$ and determine the expansion coefficients *α*_*m*_ using the DIIS method, setting *n*_0_ = 10. Finally, we solve (Eq. 21) and use the resulting *U* to evaluate (Eq. 17).

### 2.3 Computational Details

All calculations have been performed with a locally modified development version of ADF2020 using the implementation as described Förster and Visscher (2020) and using the updated imaginary frequency grids as described in Förster and Visscher (2021).

#### 2.3.1 GW100

We use the same structures as in for our previous benchmarks (Förster and Visscher, 2020; Förster and Visscher, 2021). We use the non-augmented TZ3P and QZ6P basis sets described in Förster and Visscher (2021). Complete basis set (CBS) limit extrapolated results are obtained as described in Förster and Visscher (2021). In all calculations, we set the numericalQuality key to Good. Exceptions are a few systems for which we observed inconsistencies with the Good fit set: For Pentasilane, Na_2_, Na_4_, and Na_6_, we used the Excellent fit set, and for the nucleobases we used the VeryGood fitset. We used 32 imaginary time and 32 imaginary frequency points each [We refer to the explanations in the appendix of Förster and Visscher (2021)]. For all TZ3P calculations, we set Dependency Bas = 1e−3 and for QZ6P we set Dependency Bas = 5e−3 in the AMS input as described in Förster and Visscher (2020). All calculations using augmented basis sets (aug-TZ3P and aug-QZ6P) have been performed in the same way, but using the Excellent auxiliary fit set and numericalQuality VeryGood. No relativistic effects have been taken into account.

#### 2.3.2 DNA Fragments

The structures of the DNA fragments have been taken from Doser et al. (2009). We performed *qsGW* calculations using the TZ2P (Van Lenthe and Baerends, 2003), TZ3P and QZ6P basis sets, starting from a PBE0 (Adamo and Barone, 1999; Ernzerhof and Scuseria, 1999) initial guess. We set the numerical quality to VeryGood, but used the Good fitset, with the exception of the QZ6P calculations were we also used the VeryGood fitset. We also set MBPT. ThresholdQuality = Normal. In Förster and Visscher (2020) we have shown that these thresholds are sufficient to converge quasi-particle energies within a few 10 meV. 16 grid points in imaginary time and imaginary frequency have been used. Solvent effects have been accounted for exclusively on the KS level using the conductor like screening model (COSMO) (Klamt and Schüürmann, 1993; Klamt, 1995; Klamt and Jonas, 1996) as implemented in ADF (Pye and Ziegler, 1999) using the BLYP (Becke, 1988; Lee et al., 1988; Miehlich et al., 1989) functional with D3 dispersion correction (Grimme et al., 2010) with Becke-Johnson damping (Grimme et al., 2011) and the TZ2P basis set. Numericalquality Good has been used. The solvent correction Δ*E*_*s*_ is then obtained as $\Delta E_{s}=E_{s}^{\left(+\right)}-E_{s}^{\left(0\right)}$, i.e. as the difference between the solvent contributions to the bonding energies of the oxidized species and the neutral species both at the equilibrium geometry of the neutral species.

## 3 Results

### 3.1 Benchmarks

#### 3.1.1 Comparison of Exchange-Correlation Potentials in qs*GW*

**FIGURE 1:**
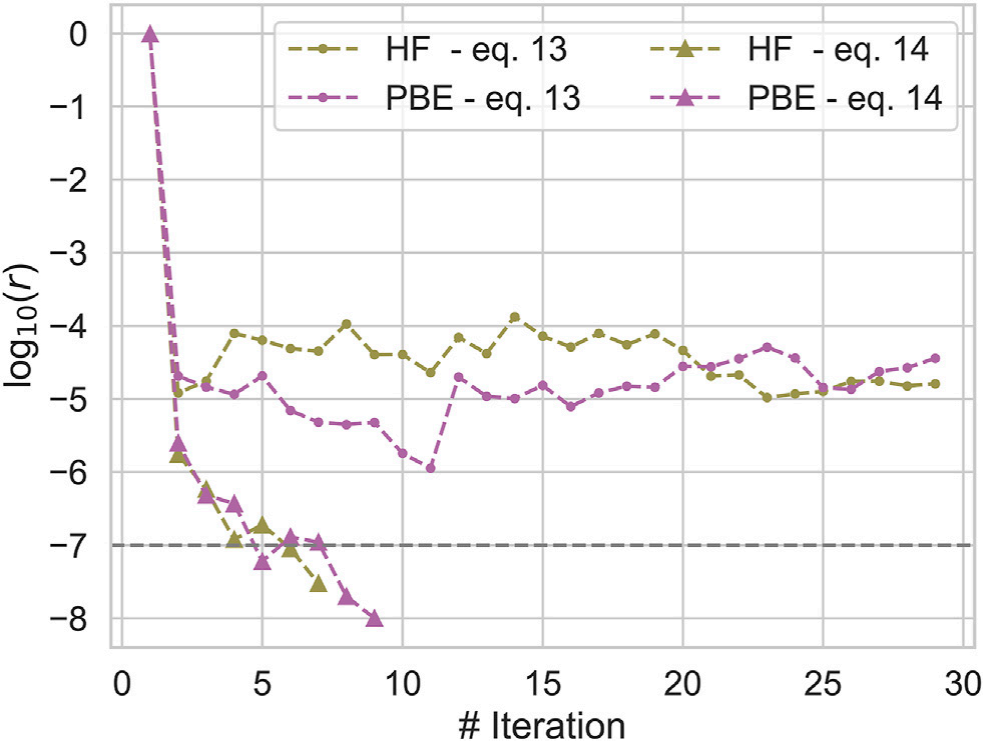
Convergence of the qs*GW* SCF for Methane for different initial guesses and constructions of the correlation potential.  log _10_ *r*, *r* defined in Eq. 26, is plotted against the number of iterations.

We already noticed in section 2 that the correlated part of the exchange-correlation potential of qs*GW* can be defined in different ways. Here we compare the two most common ways to construct this quantity (Kotani et al., 2007; Shishkin et al., 2007; Shishkin and Kresse, 2007; Kaplan et al., 2016) (Eq. 12 and Eq. 13) for a subset of molecules from the GW100 database. The data is shown in the supporting information and shows that the exchange-correlation potential obtained from (Eq. 12) is significantly harder to converge than the one from (Eq. 13). An example of the convergence behaviour of both variants is shown in Figure 1. Figure 1 plots log _10_ *r* with *r* defined in Eq. 26 against the number of iterations with two different initial guesses for Methane. We see, that using (Eq. 13), the SCF rapidly converges towards a fixed point, while log _10_ *r* always remains much larger than −6 for (Eq. 12). On the other hand, for the 10 converged calculations differences in the final QP energies are small; for both, IPs and EAs, both variants differ by only 20 meV on average, i. e the error introduced by averaging over the off-diagonal elements of the self-energy are small. For this reason, we decided to use the correlation potential as defined in (Eq. 13) in all subsequent calculations.

#### 3.1.2 Self Consistent Field Convergence

Next, we comment on the convergence of the qs*GW* SCF procedure. To this end, we compare IPs and electron affinities (EA) for the molecules in the GW100 database for 3 different starting points, PBE (Perdew et al., 1996a; Perdew et al., 1996b), PBE0, and HF. At self-consistency, the QP energies should be independent from the initial guess and their differences will thus provide information about the obtained convergence of the QP energies for a given *ε*_*SCF*_. In all calculations we set *ε*_*SCF*_ = 1*e* ^−7^ and restrict all calculations to a maximum of 30 iterations.

Independent of the starting point, we could not reach convergence for Mgo, BeO, BN, Cu_2_, and CuCN with our DIIS implementation. Employing a linear mixing procedure as implemented in Wilhelm et al. (2021) with *α* = 0.35 we could reach convergence for these systems, albeit with a large number of iterations. These systems are problematic for GW approaches since the single the spectral weight of the single excited electron is distributed between multiple peaks (Govoni and Galli, 2018). qs*GW* relies on the validity of the single QP picture. In situations, in which the quasi-particle equations might have multiple solutions (Govoni and Galli, 2018; Golze et al., 2019) corresponding to the same non-interacting state, different solutions may be found in different iterations of the qs*GW* SCF procedure. qs*GW* should select the solution with largest QP weight (Ismail-Beigi, 2017) but in situations where there are at least two solutions with (almost) equal QP weight, the “physical” solution might change in each iteration. In such cases, the DIIS algorithm tries to minimize the residual SCF error by interpolating between different solutions and no fixed point of the map (Eq. 20) is found. On the other hand, linear mixing results in a smooth but slow convergence pattern, if only *α* is chosen small enough to make sure that in all iterations the same solution is found. We do not know, how to best solve this issue but we do not consider it to be a major concern as such convergence problems are only encountered for systems in which the single QP picture is not valid. This then merely signals that qs*GW* is not an appropriate level of theory.

**FIGURE 2:**
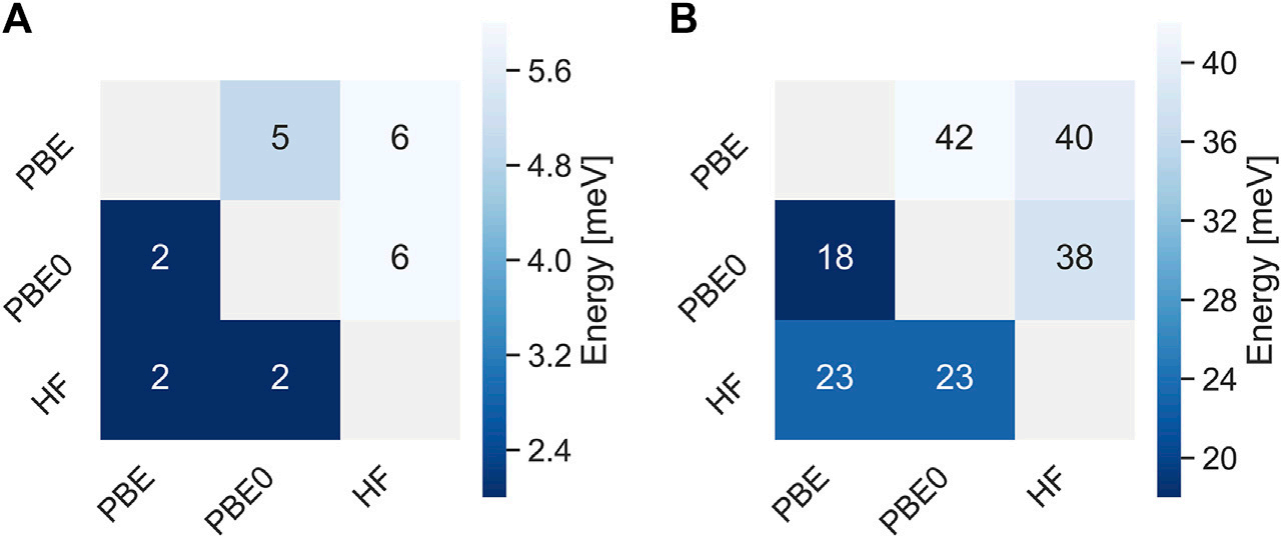
Mean absolute deviations **(A)** and maximum absolute deviations **(B)** of qsGW IPs (upper triangle) and EAs (lower triangle) obtained with different initial guesses for the GW100 database. All values are in meV.

**FIGURE 3:**
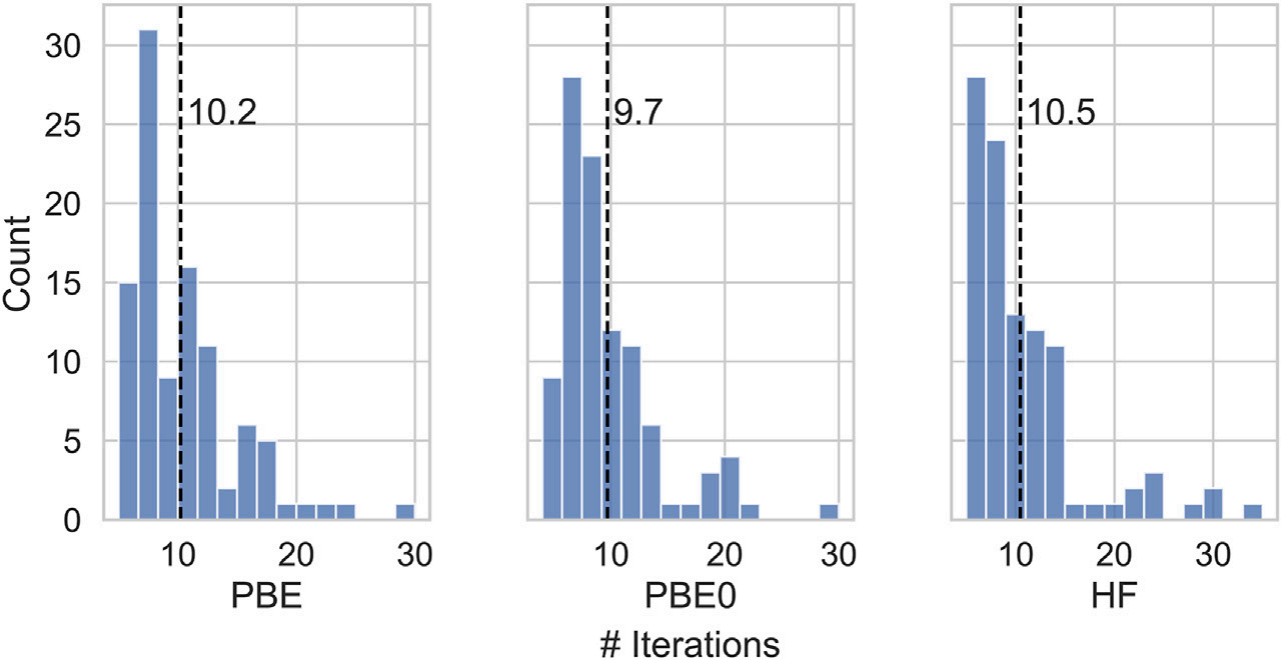
Number of iterations needed to attain convergence of the SCF for different initial guesses.

Figure 2 shows mean absolute deviations (MAD) as well as maximum absolute deviations of the IPs and EAa obtained from different starting points. With MAD of 6 and 2 meV, respectively, EAs are better converged than IPs. Also the maximum error is about twice as small for EAs than for IPs. These differences are related to the AC procedure which gives smaller errors for unoccupied states with usually featureless self-energy matrix elements. The maximum error never exceeds 50 meV and is of the same order of magnitude than the experimental resolution of photoionization experiments (Knight et al., 2016) of the typical basis set errors of *GW* calculations after extrapolation. (Knight et al., 2016; Maggio et al., 2017; Govoni and Galli, 2018; Bruneval et al., 2020; Förster and Visscher, 2021). The distribution of iterations required for convergence is displayed in Figure 3. This includes the 5 problematic cases discussed above. The calculations on average converge in around 10 iteration, with little dependence on the initial guess.

#### 3.1.3 Comparison of Ionization Potentials for the GW100 Database

We now compare the IPs from our algorithm to the ones obtained with the TURBOMOLE code for GW100. The TURBOMOLE results have been obtained with the GTO-type def2-TZVPP basis sets. For some systems, TURBOMOLE results are not available and we exclude these from our discussion. We use the TZ3P basis sets which we have shown to give comparable results to def2-TZVP for GW100 (Förster and Visscher, 2021). However, quantitative accuracy can not be expected.

**FIGURE 4:**
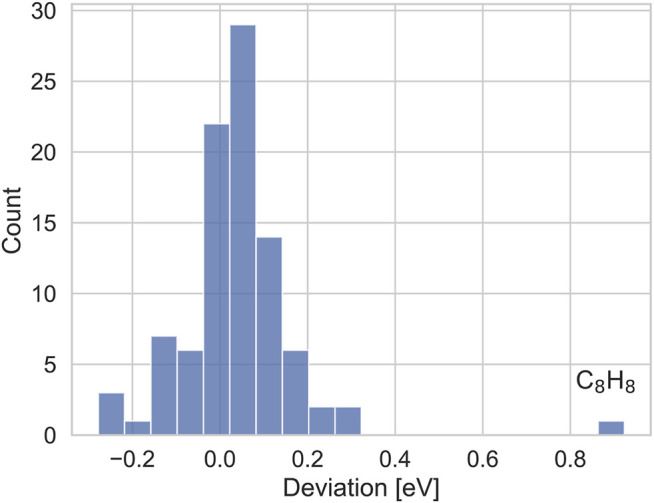
Distribution of deviations (in eV) of the IPs from TURBOMOLE and with our implementation.

The deviations to TURBOMOLE are shown in Figure 4. The average deviation between both codes is close to zero, and with one exception, for all IPs deviations are considerably smaller than 300 meV, with the deviations for the majority of systems being smaller than 100 meV. Thus, our results are qualitatively similar and deviations can be attributed to different basis set errors and different constructions of the qs*GW* exchange-correlation potential. The IP of Cyclooctatetrane is the only exception. Here, TURBOMOLE gives an IP of 9.30 eV, while the ADF IP is with 8.38 eV nearly 1 eV smaller. For different starting points, we obtained the same result within an accuracy of only a few meV, indicating that our IP is well converged. The TURBOMOLE qsGW IPs on average overestimate the CCSD(T) reference values for GW100 by Klopper and coworkers (Krause and Klopper, 2017) in the same basis set by only a little more than 100 meV, while the deviation for Cycloocatetrane is nearly 1 eV. The CCSD (T) IP for this system, is 8.35 eV, which is in very good agreement with our value. These numbers indicate that our IP is reasonable, despite the large deviation to TURBOMOLE.

Ideally, we would also like to compare our EAs against literature data, however, with only one exception (were optimized structures do not seem to be available) (Ke, 2011), we are not aware of any published EAs for molecular systems.

#### 3.1.4 Basis Set Limit Extrapolated Ionization Potentials and Electron Affinities for the GW100 Database

In the supporting information, we report CBS limit extrapolated EAs and IPs for the GW100 database. The qs*GW* QP energies seem to converge faster to the CBS limit than their *G*_0_ *W*_0_ counterparts. Going from TZ3P to QZ6P, the basis set incompleteness error reduces by 80 meV on average, while for *G*_0_ *W*_0_ *@*PBE, we found an average reduction of 130 meV (Förster and Visscher, 2021). Self-consistent approaches might converge faster than *G*_0_ *W*_0_ - Caruso et al. have already observed that sc*GW* converges faster to the CBS limit than *G*_0_ *W*_0_ (Caruso et al., 2013). For the EAs, the average differences are much larger which is also due to the many systems with negative EA in the GW100 database. For these systems CBS limit extrapolation is not reliable without adding diffuse functions. Repeating these calculations with augmented basis sets (Förster and Visscher, 2021) yields smaller differences between the aug-TZ3P and aug-QZ6P basis sets. (Förster and Visscher, 2021). In Table 1, these differences are shown for the series of linear alkanes from Methane to Butane (for more numbers we refer to the supporting information). On both the TZ and QZ level the augmented basis sets give a much higher EA. Also, the differences between aug-TZ3P and aug-QZ6P are with in between 150 and 200 meV modest, while they are huge for the non-augmented basis sets. Also the extrapolated values are much smaller using the augmented basis sets. The effect of augmentation is also profound for other systems. For example, using the non-augmented basis sets, the EA of carbontetrachloride is negative (−0.27 eV). Using the augmented basis sets, it becomes positive (0.17 eV) which is in much better agreement with experiment (0.80 ± 0.34 eV) (Staneke et al., 1995).

**TABLE 1:** Comparison of electron affinities for linear alkanes from Methane to Butane using augmented, and non-augmented basis sets.

	Non-augmented			Augmented		
Name	TZ3P	QZ6P	Extrap	Aug-TZ3P	Aug-QZ6P	Extrap
Methane	−2.30	−−1.62	−0.78	−0.79	−0.58	−0.26
Ethane	−2.27	−1.56	−0.65	−0.72	−0.57	−0.35
Propane	−2.23	−1.51	−0.56	−0.72	−0.55	−0.30
Butane	−2.24	−1.50	−0.52	−0.71	−0.55	−0.30

### 3.2 Application to DNA Fragments

Oxidation of DNA is related to genetic damage and to investigate the mechanisms behind these processes quantum chemically, electron addition and removal energies need to be computed with high accuracy. A necessary first step for such studies is the selection of appropriate model system which should represent DNA under physiological conditions as accurately as possible while still being computationally feasible. As an illustrative example how the new qsGW implementation can be used effectively in practice, we investigate the dependence of IP and EA of oligomers of Adenine-Thymine (AT) base pairs on the oligomer size.

**FIGURE 5:**
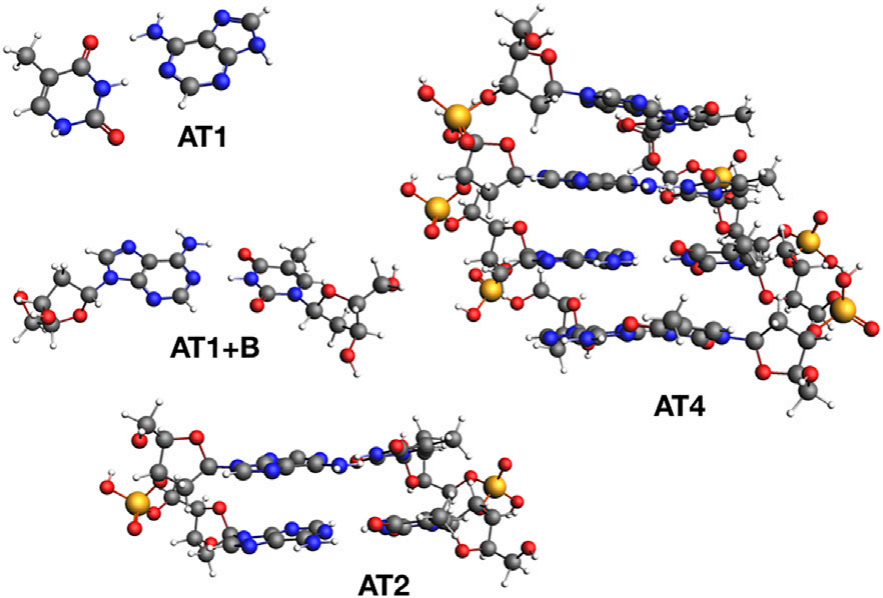
DNA model systems used in this work.

The calculated charged excitations are shown in Table 2 for different basis sets and fragment sizes between 1 and 4 AT pairs (We refer to these systems as AT*x*, were *x* denotes the number of AT base pairs). These systems are shown in Figure 5. For all fragments, we calculated the IPs with the TZ2P and TZ3P basis set with 1d1f, and 2d1f shells of polarization functions for second and third row atoms (and analogously for other atoms). We see, that going from TZ2P to TZ3P only has a small effect on the IPs and EAs, reducing the basis set incompleteness error by only a few 10 meV. These calculations with two rather similar basis sets are necessary to rule out the possibility that a result is simply an artefact of a chosen basis set. Going from TZ3P to QZ6P, the IP of the AT1+B increases by modest 60 meV, while the EA reduces by 180 meV. Based on the TZ3P and QZ6P calculations, we can estimate the QP energies at the CBS limit by extrapolation. Comparing the TZ3P results to the extrapolated ones, we find a basis set limit incompleteness error of 140 meV for the IP and of 420 meV for the EA of AT1. For AT1, we find a similar basis set limit incompleteness error of 80 meV for the IP and of 340 meV for the EA.

**TABLE 2:** Ionization potentials (IPs) and electron affinities (EAS) of DNA fragments consisting of different numbers of adenine-thymine base pairs calculated with different basis sets and contributions of solvent from ΔBLYP calculations. Extra denotes extrapolation to the CBS limit based on TZ3P and QZ6P calculations and numbers in parentheses are obtained by adding the difference between $\epsilon_{i}^{CBS}-\epsilon_{i}^{TZ3P}$ to the result obtained at the TZ3P level. Δsol. has been calculated using COSMO. All values are in eV.

	IP				EA			
Calculation	AT1	AT1+B	AT2	AT4	AT1	AT1+B	AT2	AT4
TZ2P	—	7.84	7.34	6.94	—	−0.84	−0.65	−0.45
TZ3P	8.47	7.90	7.35	6.97	−0.41	−0.80	−0.63	−0.40
QZ6P	8.50	7.96	—	—	−0.26	−0.62	—	—
Extra	8.55	8.04	(7.49)	(7.11)	0.07	-0.38	(−0.21)	(0.02)
Δsol	−1.82	-0.99	−0.52	−0.01	1.55	—	1.87	1.62
*ϵ* + Δsol	6.73	7.05	6.97	7.10	1.62	—	1.66	1.64

On standard hardware, calculations on the QZ level are not feasible for AT4 and already for AT2, the QZ calculation is cumbersome. This is not only due to the large number of diffuse AOs which make makes it difficult to exploit distance-based cut-offs (Förster and Visscher, 2020) but also due to the large auxiliary basis sets which are required to make the calculations numerically stable. However, we can estimate the CBS limit based on the differences between the QP energies at the CBS limit and the largest affordable basis set for the larger systems for the smaller fragments. This is justified with the observations made in Förster and Visscher (2020) for *G*_0_ *W*_0_ were we found the basis set incompleteness error on average to decrease with increasing system size but only to a certain extent since basis functions are localised. Based on this assumption, we correct the IPs and EAs of AT2 and AT4 on the TZ3P level by the basis set limit incompleteness error found for AT1+B. (140 and 420 meV, respectively). There is of course a small uncertainty due to the different basis set errors for AT1 and AT1+B. For the extrapolation itself, we assume the error to be rather small for the IP, since the difference between TZ3P and QZ6P are rather small. For the EAs, the error might be larger. Still, we can safely assume, that the basis set errors for AT2 and AT4 are below 100 meV.

The energy required to remove or add an electron from a DNA oligomer in vacuum is strongly size dependent: The vertical IP in vacuum decreases rapidly with increasing oligomer size, with a difference of almost 1 eV between AT1 and AT4. For the EA, a difference of 0.4 eV is found. The IPs of the solvated DNA oligomers, on the other hand, are almost independent of the number of base pairs. When an electron is removed from the oligomer, the surrounding cloud of electrons stabilizes the resulting hole. Increasing the oligomer size thus reduces the IP potential since the hole becomes more and more stabilized. In the aqueous environment, the solvent plays the same role and consequently, the inclusion of water via the COSMO effectively compensates for the effect of the DNA environment. Of course, the comparison is slightly skewed since the DNA environment and the solvent are not treated at the same level of theory. However, there is some evidence that COSMO and other polarizable continuum models are fairly accurate in describing the dielectric screening properties of water (Deglmann and Schenk, 2012).

The IP of AT1+B, AT2, and AT4, all agree within 130 meV. In light of possible basis set errors and errors of the qsGW method itself, the difference is well within the error margin of our method. Only for AT1 we obtain a significantly lower IP, which indicates that the DNA backbone apparently plays an important role in stabilizing ionized DNA oligomers. For the EAs, we arrive at the same conclusion. The differences between the considered systems are even smaller, the aqueous EAs of AT1, AT2 and AT4 being with 1.62, 1.66, and 1.64 eV in excellent agreement. Recently, Pluhařová et al. (2011), Pluhařová et al. (2013), Pluhařová et al. (2015) also concluded that the effect of the DNA environment on the IPs of individual aqueous nucleobases seems to be modest. On the BMK (Boese and Martin, 2004)/6–31G* level of theory, they obtained an IP of 7.24 eV for a fragment of 2 solvated AT base pairs including backbone from the Dickerson dodecamer, but for the isoltaed AT base pair, they obtained and IP of 7.58 eV. The first number is in good agreement with ours, while the second one differs from our result for AT1 by almost 1 eV. However, the difference of only 340 meV between both fragments is of the same order as our difference between the IPs of AT1 and AT2 of 260 meV. Thus, our conclusions regarding the role of the explicit inclusion of the DNA environment on the calculated IPs are very similar.

Finally, we shortly discuss the compute times of the qs*GW* calculations for the DNA fragments. A detailed timing analysis for the evaluation of the self-energy in ADF has already been performed in Förster and Visscher (2020). The asymptotic scaling of qs*GW* will be the same as for *G*_0_ *W*_0_: The only additional cubic step is the diagonalization of the Hamiltonian in each iteration. The LU factorization of each of the *N*_*ω*_ *N*_*fit*_ × *N*_*fit*_ matrices in each iteration to calculate the screened interaction (Förster and Visscher, 2020) requires roughly $\frac{2}{3}N_{fit}^{3}$ FLOPS, while the dominant step in the single diagonalization of the *N*_*bas*_ × *N*_*bas*_ matrix in each iteration requires $\frac{4}{3}N_{bas}^{3}$ FLOPS. Since we have *N*_*bas*_ ≈ 5 × *N*_*fit*_ in a typical calculation, the compute time for diagonalization is negligible. Of course, a qsGW calculation requires multiple iterations and is consequently slower than a *G*_0_ *W*_0_ calculation. For the DNA fragments, all calculations required between 6 and 8 iterations to converge. This is considerably faster than the average number of iterations found for GW100, where we have already observed that convergence is typically faster for organic systems. We have set the converge threshold for all calculations in this section to *log*_10_ (*ε*_*SCF*_) = −8, as opposed to −7 for GW100. However, the increasing sparsity of *G*_0_ (*τ* → 0^+^) and *G*_0_ (*τ* → 0^−^) with increasing system size is also responsible for this fast convergence.

The largest calculation here is the one for AT4 using the TZ3P basis set. The system has 260 atoms and 1,220 electrons. We used 6,374 MOs and 33,678 auxiliary fit functions. The calculation took 6 iterations to converge and has been performed on 16 cores of a single Dual AMD EPYC 7302@3.0GHz, 2x RTX2070 machine with 256 GB of memory. On average, a single iteration took a little more than 15 h, or 243 core hours.

## 4 Conclusion

As opposed to *GW* calculations with diagonal self-energy, qsGW is a general, parameter-free, and starting point independent method for the calculation of QP energies. While qsGW is known to severely overestimate band gaps and IPs in three-dimensional (3D) materials (Shishkin et al., 2007; Tal et al., 2021) there is evidence that qsGW is more accurate for molecules (Caruso et al., 2016; Kaplan et al., 2016). In canonical implementations, qsGW is usually a magnitude slower than evGW (Gui et al., 2018) and so far, low-order scaling implementations for molecular systems have focused on diagonal approximations to *GW* (Wilhelm et al., 2018, Wilhelm et al., 2021; Förster and Visscher, 2020; Duchemin and Blase, 2021). To fill this gap, we have presented a low-order scaling implementation of qs*GW* for molecular systems and demonstrated its accuracy and robustness. In a proof-of-principle application to DNA fragments we have showcased the capabilities of the new implementation for systems of practical interest (Pluhařová et al., 2015; Balanikas et al., 2020). We have shown, that IPs and EAs of the considered DNA fragments in vacuum are strongly size-dependent. Upon taking into account the effect of the aqueous environment, the QP energies become almost independent of the system size. This confirms the results of previous DFT studies. (Pluhařová et al., 2015, Pluhařová et al., 2013). For the largest of the considered fragments with 1,220 electrons, the respective qsGW calculation with more than 6,300 spherical AOs converged within 6 iterations in less than 4 days on a single compute node with 16 cores.

All in all, the herein presented implementation is a necessary stepping stone towards accurate *ab initio* studies of the spectroscopic properties of large molecules in realistic environments, relevant to organic optoelectronics or biochemistry. To be able to also study optical properties of large systems, it needs to be combined with an implementation of the BSE formalism. Our implementation does not allow to take into account solvent effects directly. In the present work, we have done that *via* a ΔDFT calculation and obtained consistent results. However, it would be desirable to take into account environmental effects more directly by combining qsGW with COSMO (or a PCM) (Duchemin et al., 2016; Li et al., 2018) and/or molecular mechanics calculations (Tirimbò et al., 2020a; Tirimbò et al., 2020b).

Another issue in practice is the slow convergence of the QP energies to the CBS limit. This is especially true for algorithms like the present one which exploit sparsity in the AO basis. It is encouraging that this convergence is seemingly faster than for qsGW than *G*_0_ *W*_0_. This doesn’t eliminate the need for basis set limit extrapolation, but the extrapolation schemes become more reliable with decreasing basis set error. Basis set errors for large systems can also be accurately estimated based on results for smaller, chemically similar systems, as exemplified in this work.

## Data Availability

The original contributions presented in the study are included in the article/Supplementary Material, further inquiries can be directed to the corresponding author.

## Footnotes

1 We experimented with different residuals: In the implementation used in this work, we have used

$$r^{\left(n+1\right)}=\left[P^{\left(n+1\right)}+Q^{\left(n+1\right)}\right]-\left[P^{\left(n\right)}+Q^{\left(n\right)}\right].$$

Here, *Q* is defined like *P*, but with the summation spanning the virtual orbital space,

$$Q_{\mu \nu }=\overset{N_{virt}}{\underset{a}{\sum }}b_{\mu a}{\left[b^{†}\right]}_{a\nu }.$$

This was based on the intuitive assumption that convergence could be improved by including information about the virtual orbitals in the residual. However, note that $S^{-1}=\frac{1}{2}P+Q$, so that we obtain $P+Q=\frac{1}{2}P+S^{-1}$. Apart from the factor of 1/2 (Eq. 26) is therefore completely equivalent to this expression. We thank one of the reviewers for pointing this out
