# Supplementary material for: Low-Order Scaling Quasiparticle Self-Consistent GW for Molecules
Source: Front Chem. 2021 Sep 3;9:736591. doi: 10.3389/fchem.2021.736591 (PMC8446457; doi:10.3389/fchem.2021.736591)
Supplement: Supplementary file 1 [file DataSheet1.pdf]

Supplementary Material

1 COMPARISON OF METHODS TO CONSTRUCT THE HAMILTONIAN OF QSGW

Table S1: Comparison of IPs and EAs combined with different schemes to construct the correlation part of the qsGW Hamiltonian. KSF1 and KSF2 refer to eq. 12 and eq. 13 in the main text, respectively. All values are in eV and the calculations have been performed with a PBE0 initial guess and using the TZ3P basis set.

name	KSF1		KSF2	
	IP	EA	IP	EA
Helium	24.262	−8.708	24.322	−8.721
Neon	21.773	−11.335	21.743	−11.351
Hydrogen	16.488	−3.059	16.488	−3.070
Lithiumdimer	5.278	0.141	5.258	0.118
Nitrogen	15.779	−3.167	15.743	−3.227
Fluorine	16.228	0.013	16.208	0.054
Lithiumhydride	8.124	0.093	8.094	0.095
Borane	13.560	−0.581	13.558	−0.622
Magnesiumfluoride	14.104	0.122	14.107	0.121
Carbondioxide	14.045	−4.153	14.011	−4.176

2 GW100

The following tables contain all calculated values for the GW100 database.

2.1 TZ3P Calculations for different starting points

2.1.1 PBE

Table S2: qsGW@PBE/TZ3P ionization potentials and electron affinities, together with the number of imaginary time and imaginary frequency points used in each iteration, and the number of iterations until convergence

	Name	IP	EA	nIter	nTime	nFreq
1	Helium	24.322	−8.721	6	7	7
2	Neon	21.742	−11.351	11	12	12
3	Argon	15.485	−8.765	9	15	15
4	Krypton	13.943	−7.422	9	18	18
5	Xenon	12.271	−5.597	16	27	28
6	Hydrogen	16.488	−3.070	8	8	8
7	Lithiumdimer	5.256	0.118	9	13	13
8	Sodiumdimer	4.997	0.217	10	16	16
9	Sodiumtetramer	4.254	0.475	8	17	17
10	Sodiumhexamer	4.396	0.469	6	16	16
11	Dipotassium	4.041	0.289	8	20	20
12	Dirubidium	3.838	0.282	10	27	29
13	Nitrogen	15.743	−3.227	6	13	13
14	Phosphorusdimer	10.269	0.128	10	16	16

Continued on next page

	Name	IP	EA	nIter	nTime	nFreq
15	Arsenicdimer	9.653	0.591	10	20	20
16	Fluorine	16.203	0.047	8	13	13
17	Chlorine	11.489	0.209	7	18	18
18	Bromine	10.663	1.008	9	20	20
19	Iodine	9.607	1.279	8	27	29
20	Methane	14.562	−2.297	10	13	13
21	Ethane	12.977	−2.267	7	13	13
22	Propane	12.317	−2.230	7	14	14
23	Buthane	11.834	−2.238	7	13	13
24	Ethylene	10.538	−2.432	7	14	14
25	Acetylene	11.295	−2.441	6	14	14
26	Tetracarbon	11.176	1.972	7	15	15
27	Cyclopropane	10.977	−2.423	7	14	14
28	Benzene	9.235	−1.831	6	14	14
29	Cyclooctatetraene	8.377	−0.888	5	15	15
30	Cyclopentadiene	8.612	−1.851	6	14	14
31	Vynilfluoride	10.563	−2.295	10	14	14
32	Vynilchloride	10.073	−2.157	7	16	16
33	Vynilbromide	9.225	−2.036	7	20	20
34	Vyniliodide	9.372	−1.454	8	27	28
35	Carbontetrafluoride	16.746	−2.270	10	13	13
36	Carbontetrachloride	11.664	−0.846	7	16	16
37	Carbontetrabromide	10.604	0.494	7	20	20
38	Carbontetraiodide	9.377	1.474	11	27	29
39	Silane	13.065	−1.826	12	16	16
40	Germane	12.746	−1.588	13	20	20
41	Disilane	10.865	−1.609	7	16	16
42	Pentasilane	9.478	−0.833	6	16	16
43	Lithiumhydride	8.094	0.095	18	12	12
44	Potassiumhydride	6.246	0.130	18	18	18
45	Borane	13.558	−0.622	9	12	12
46	Diborane6	12.609	−1.469	7	12	12
47	Amonia	11.032	−1.878	13	15	15
48	Hydrogenazide	10.749	−1.725	11	15	15
49	Phosphine	10.628	−1.576	7	17	17
50	Arsine	10.493	−1.542	12	19	19
51	Hydrogensulfide	10.312	−1.658	7	16	16
52	Hydrogenfluoride	16.397	−1.637	14	13	13
53	Hydrogenchloride	12.615	−1.654	8	17	17
54	Lithiumfluoride	11.745	0.168	14	14	14
55	Magnesiumfluoride	14.133	0.122	12	14	14
56	Titaniumfluoride	15.729	0.582	16	16	16
57	Aluminumtrifluoride	15.754	−0.527	13	15	15
58	Fluoroborane	11.078	−1.664	15	14	14
59	Sulfertetrafluoride	13.121	−0.846	9	17	17
60	Potassiumbromide	8.282	0.464	9	21	21
61	Galliummonochloride	9.820	−0.129	15	20	20
62	Sodiumchloride	9.106	0.539	9	17	17
63	Magnesiumchloride	11.676	0.216	7	18	18
64	Aluminumtriiodide	9.875	0.270	8	26	26
65	Boronnitride	11.694	3.139	20	16	16
66	Hydrogencyanide	13.606	−2.168	12	14	14
67	Phosphorusmononitride	11.902	−0.419	17	15	15
68	Hydrazene	9.993	−1.706	11	15	15
69	Formaldehyde	11.180	−1.642	11	15	15
70	Methanol	11.417	−2.002	16	15	15
71	Ethanol	11.071	−1.972	7	15	15
72	Acetaldehyde	10.578	−1.909	11	16	16
73	Ethoxyethane	10.207	−2.183	7	16	16
74	FormicAcid	11.812	−1.955	12	14	14
75	Hydrogenperoxide	11.968	−1.813	11	16	16

Continued on next page

	Name	IP	EA	nIter	nTime	nFreq
76	Water	12.882	−1.638	17	16	16
77	Carbondioxide	14.014	−4.175	9	16	16
78	Carbondisulfide	9.895	−0.430	13	16	16
79	Carbonoxysulfide	11.183	−1.925	12	17	17
80	Carbonoxyselenide	10.550	−1.451	10	20	20
81	Carbonmonoxide	14.449	−1.307	13	16	16
82	Ozon	13.143	1.892	17	15	15
83	Sulferdioxide	12.617	0.527	10	18	18
84	Berylliummonoxide	10.224	1.931	30	16	16
85	Magnesiummonoxide	8.077	1.585	19	17	17
86	Tuloene	8.881	−1.754	5	15	15
87	Ethybenzene	8.781	−1.773	5	15	15
88	Hexafluorobenzene	10.183	−0.901	6	14	14
89	Phenol	8.735	−1.633	6	16	16
90	Aniline	8.030	−1.787	6	15	15
91	Pyridine	9.653	−1.237	7	15	15
92	Guanine	8.101	−1.167	6	16	16
93	Adenine	8.386	−1.233	6	15	15
94	Cytosine	8.965	−0.936	7	16	16
95	Thymine	9.277	−0.760	7	16	16
96	Uracil	9.676	−0.703	7	15	15
97	Urea	10.462	−1.481	8	15	15
98	Silverdimer	6.987	0.492	16	26	26
99	Copperdimer	7.611	0.351	23	21	21
100	Coppercyanide	10.997	0.942	24	20	20

2.1.2 PBE0

Table S3: qsGW@PBE0/TZ3P ionization potentials and electron affinities, together with the number of imaginary time and imaginary frequency points used in each iteration, and the number of iterations until convergence

	Name	IP	EA	nIter	nTime	nFreq
1	Helium	24.322	−8.721	6	7	7
2	Neon	21.743	−11.351	13	12	12
3	Argon	15.485	−8.766	6	15	15
4	Krypton	13.943	−7.422	9	18	18
5	Xenon	12.271	−5.596	10	26	26
6	Hydrogen	16.488	−3.070	8	8	8
7	Lithiumdimer	5.258	0.118	9	13	13
8	Sodiumdimer	4.995	0.215	12	18	18
9	Sodiumtetramer	4.252	0.472	8	17	17
10	Sodiumhexamer	4.388	0.471	6	17	17
11	Dipotassium	4.041	0.290	6	20	20
12	Dirubidium	3.836	0.281	9	27	29
13	Nitrogen	15.743	−3.227	6	13	13
14	Phosphorusdimer	10.268	0.128	7	16	16
15	Arsenicdimer	9.649	0.593	10	20	20
16	Fluorine	16.208	0.054	10	13	13
17	Chlorine	11.487	0.210	6	18	18
18	Bromine	10.672	1.009	9	20	20
19	Iodine	9.605	1.278	8	27	29
20	Methane	14.564	−2.297	7	13	13
21	Ethane	12.976	−2.268	7	13	13
22	Propane	12.321	−2.229	6	14	14
23	Buthane	11.850	−2.237	5	13	13
24	Ethylene	10.539	−2.433	6	15	15
25	Acetylene	11.295	−2.441	6	14	14
26	Tetracarbon	11.204	1.973	6	16	16
27	Cyclopropane	10.979	−2.423	7	14	14
28	Benzene	9.242	−1.824	5	14	14
29	Cyclooctatetraene	8.379	−0.895	5	15	15
30	Cyclopentadiene	8.624	−1.846	5	14	14
31	Vynilfluoride	10.582	−2.296	10	15	15
32	Vynilchloride	10.076	−2.161	7	16	16
33	Vynilbromide	9.267	−2.032	8	20	20
34	Vyniliodide	9.371	−1.452	8	27	28
35	Carbontetrafluoride	16.745	−2.270	10	13	13
36	Carbontetrachloride	11.665	−0.844	10	16	16
37	Carbontetrabromide	10.609	0.476	10	20	20
38	Carbontetraiodide	9.380	1.489	12	27	29
39	Silane	13.065	−1.826	7	16	16
40	Germane	12.742	−1.590	12	20	20
41	Disilane	10.864	−1.610	7	16	16
42	Pentasilane	9.473	−0.829	7	16	16
43	Lithiumhydride	8.094	0.095	11	12	12
44	Potassiumhydride	6.258	0.129	13	19	19
45	Borane	13.558	−0.622	7	12	12
46	Diborane6	12.609	−1.469	6	12	12
47	Amonia	11.033	−1.877	13	15	15
48	Hydrogenazide	10.750	−1.725	11	15	15
49	Phosphine	10.628	−1.577	7	17	17
50	Arsine	10.492	−1.542	12	19	19
51	Hydrogensulfide	10.312	−1.658	7	16	16
52	Hydrogenfluoride	16.403	−1.636	12	13	13
53	Hydrogenchloride	12.614	−1.654	9	17	17

Continued on next page

	Name	IP	EA	nIter	nTime	nFreq
54	Lithiumfluoride	11.774	0.167	20	14	14
55	Magnesiumfluoride	14.107	0.121	14	14	14
56	Titaniumfluoride	15.732	0.581	14	16	16
57	Aluminumtrifluoride	15.768	−0.526	9	15	15
58	Fluoroborane	11.076	−1.667	16	14	14
59	Sulfertetrafluoride	13.125	−0.848	9	17	17
60	Potassiumbromide	8.289	0.464	9	21	21
61	Galliummonochloride	9.807	−0.128	13	20	20
62	Sodiumchloride	9.105	0.539	8	17	17
63	Magnesiumchloride	11.678	0.217	6	18	18
64	Aluminumtriiodide	9.882	0.274	6	27	27
65	Boronnitride	11.682	3.141	17	15	15
66	Hydrogencyanide	13.602	−2.166	11	14	14
67	Phosphorusmononitride	11.902	−0.419	30	15	15
68	Hydrazene	9.999	−1.706	11	16	16
69	Formaldehyde	11.203	−1.637	10	15	15
70	Methanol	11.426	−2.002	9	16	16
71	Ethanol	11.073	−1.972	8	15	15
72	Acetaldehyde	10.577	−1.908	10	16	16
73	Ethoxyethane	10.198	−2.183	7	15	15
74	FormicAcid	11.814	−1.955	9	16	16
75	Hydrogenperoxide	11.965	−1.814	10	16	16
76	Water	12.887	−1.638	12	16	16
77	Carbondioxide	14.011	−4.176	18	14	14
78	Carbondisulfide	9.894	−0.432	11	18	18
79	Carbonoxysulfide	11.182	−1.927	18	17	17
80	Carbonoxyselenide	10.535	−1.453	8	20	20
81	Carbonmonoxide	14.449	−1.307	21	16	16
82	Ozon	13.143	1.890	9	15	15
83	Sulferdioxide	12.617	0.527	10	16	16
84	Berylliummonoxide	10.229	1.931	23	16	16
85	Magnesiummonoxide	8.101	1.583	20	16	16
86	Tuloene	8.876	−1.757	5	15	15
87	Ethybenzene	8.781	−1.788	4	15	15
88	Hexafluorobenzene	10.199	−0.899	8	14	14
89	Phenol	8.737	−1.630	6	16	16
90	Aniline	8.039	−1.781	5	15	15
91	Pyridine	9.654	−1.230	5	15	15
92	Guanine	8.100	−1.164	6	16	16
93	Adenine	8.391	−1.229	5	15	15
94	Cytosine	8.968	−0.935	6	16	16
95	Thymine	9.278	−0.760	7	15	15
96	Uracil	9.653	−0.706	9	15	15
97	Urea	10.461	−1.479	9	16	16
98	Silverdimer	6.997	0.493	10	26	26
99	Copperdimer	7.610	0.351	19	21	21
100	Coppercyanide	10.997	0.943	21	20	20

2.1.3 Hartree-Fock

Table S4: qsGW@Hartree-Fock/TZ3P ionization potentials and electron affinities, together with the number of imaginary time and imaginary frequency points used in each iteration, and the number of iterations until convergence

	Name	IP	EA	nIter	nTime	nFreq
1	Helium	24.322	−8.721	6	7	7
2	Neon	21.745	−11.350	13	12	12
3	Argon	15.485	−8.766	5	16	16
4	Krypton	13.938	−7.422	9	19	19
5	Xenon	12.271	−5.597	8	27	28
6	Hydrogen	16.488	−3.070	8	8	8
7	Lithiumdimer	5.258	0.116	9	13	13
8	Sodiumdimer	4.993	0.213	11	18	18
9	Sodiumtetramer	4.261	0.466	7	16	16
10	Sodiumhexamer	4.386	0.471	6	17	17
11	Dipotassium	4.041	0.287	10	20	20
12	Dirubidium	3.837	0.280	9	27	29
13	Nitrogen	15.743	−3.227	7	13	13
14	Phosphorusdimer	10.268	0.128	8	16	16
15	Arsenicdimer	9.649	0.591	10	20	20
16	Fluorine	16.210	0.055	7	13	13
17	Chlorine	11.489	0.210	7	18	18
18	Bromine	10.679	1.009	8	20	20
19	Iodine	9.605	1.279	6	27	27
20	Methane	14.567	−2.297	8	13	13
21	Ethane	12.970	−2.269	6	13	13
22	Propane	12.283	−2.231	6	13	13
23	Buthane	11.861	−2.237	6	14	14
24	Ethylene	10.537	−2.430	8	14	14
25	Acetylene	11.299	−2.440	6	14	14
26	Tetracarbon	11.176	1.974	6	15	15
27	Cyclopropane	10.973	−2.423	6	14	14
28	Benzene	9.246	−1.819	6	14	14
29	Cyclooctatetraene	8.381	−0.890	6	15	15
30	Cyclopentadiene	8.604	−1.848	6	14	14
31	Vynilfluoride	10.562	−2.296	10	14	14
32	Vynilchloride	10.076	−2.162	8	16	16
33	Vynilbromide	9.266	−2.031	8	20	20
34	Vyniliodide	9.371	−1.452	8	27	28
35	Carbontetrafluoride	16.744	−2.270	12	13	13
36	Carbontetrachloride	11.665	−0.843	10	16	16
37	Carbontetrabromide	10.602	0.487	10	20	20
38	Carbontetraiodide	9.380	1.489	11	26	26
39	Silane	13.066	−1.826	7	16	16
40	Germane	12.746	−1.590	8	20	20
41	Disilane	10.865	−1.613	6	16	16
42	Pentasilane	9.479	−0.831	5	15	15
43	Lithiumhydride	8.094	0.095	8	12	12
44	Potassiumhydride	6.242	0.127	11	18	18
45	Borane	13.555	−0.623	6	12	12
46	Diborane6	12.609	−1.469	6	12	12
47	Amonia	11.042	−1.877	11	14	14
48	Hydrogenazide	10.742	−1.726	14	14	14
49	Phosphine	10.628	−1.576	7	17	17
50	Arsine	10.494	−1.542	14	19	19
51	Hydrogensulfide	10.313	−1.658	7	16	16
52	Hydrogenfluoride	16.386	−1.637	12	13	13
53	Hydrogenchloride	12.613	−1.654	6	17	17

Continued on next page

	Name	IP	EA	nIter	nTime	nFreq
54	Lithiumfluoride	11.748	0.167	13	14	14
55	Magnesiumfluoride	14.115	0.122	12	14	14
56	Titaniumfluoride	15.743	0.578	19	16	16
57	Aluminumtrifluoride	15.760	−0.527	8	15	15
58	Fluoroborane	11.077	−1.664	13	14	14
59	Sulfertetrafluoride	13.122	−0.843	9	18	18
60	Potassiumbromide	8.286	0.464	9	21	21
61	Galliummonochloride	9.819	−0.131	12	20	20
62	Sodiumchloride	9.105	0.542	10	17	17
63	Magnesiumchloride	11.677	0.216	9	17	17
64	Aluminumtriiodide	9.884	0.275	6	27	27
65	Boronnitride	11.693	3.140	35	16	16
66	Hydrogencyanide	13.603	−2.167	14	14	14
67	Phosphorusmononitride	11.904	−0.419	21	15	15
68	Hydrazene	10.005	−1.705	12	14	14
69	Formaldehyde	11.199	−1.637	18	15	15
70	Methanol	11.423	−2.002	8	16	16
71	Ethanol	11.076	−1.972	6	15	15
72	Acetaldehyde	10.580	−1.908	10	15	15
73	Ethoxyethane	10.204	−2.184	6	15	15
74	FormicAcid	11.823	−1.955	14	16	16
75	Hydrogenperoxide	11.965	−1.814	6	16	16
76	Water	12.890	−1.638	13	16	16
77	Carbondioxide	14.012	−4.177	12	14	14
78	Carbondisulfide	9.895	−0.430	21	16	16
79	Carbonoxysulfide	11.185	−1.926	13	17	17
80	Carbonoxyselenide	10.543	−1.449	12	20	20
81	Carbonmonoxide	14.442	−1.307	23	15	15
82	Ozon	13.144	1.891	30	15	15
83	Sulferdioxide	12.618	0.528	14	16	16
84	Berylliummonoxide	10.195	1.933	30	17	17
85	Magnesiummonoxide	8.101	1.583	28	16	16
86	Tuloene	8.869	−1.761	6	14	14
87	Ethybenzene	8.801	−1.765	5	14	14
88	Hexafluorobenzene	10.196	−0.900	8	14	14
89	Phenol	8.731	−1.633	6	15	15
90	Aniline	8.031	−1.782	6	15	15
91	Pyridine	9.651	−1.236	6	15	15
92	Guanine	8.116	−1.144	8	16	16
93	Adenine	8.364	−1.247	6	16	16
94	Cytosine	8.952	−0.943	8	15	15
95	Thymine	9.281	−0.764	8	16	16
96	Uracil	9.646	−0.704	13	16	16
97	Urea	10.472	−1.474	11	15	15
98	Silverdimer	6.988	0.492	15	26	26
99	Copperdimer	7.607	0.352	23	21	21
100	Coppercyanide	10.998	0.943	23	20	20

Table S5: Comparison of ionization potentials and electron affinities obtained form the three different starting points. All values are in eV.

		IP			EA		
	Name	PBE	PBE0	Hartree-Fock	PBE	PBE0	Hartree-Fock
1	Helium	24.322	24.322	24.322	−8.721	−8.721	−8.721
2	Neon	21.742	21.743	21.745	−11.351	−11.351	−11.350
3	Argon	15.485	15.485	15.485	−8.765	−8.766	−8.766
4	Krypton	13.943	13.943	13.938	−7.422	−7.422	−7.422
5	Xenon	12.271	12.271	12.271	−5.597	−5.596	−5.597
6	Hydrogen	16.488	16.488	16.488	−3.070	−3.070	−3.070
7	Lithiumdimer	5.256	5.258	5.258	0.118	0.118	0.116
8	Sodiumdimer	4.997	4.995	4.993	0.217	0.215	0.213
9	Sodiumtetramer	4.254	4.252	4.261	0.475	0.472	0.466
10	Sodiumhexamer	4.396	4.388	4.386	0.469	0.471	0.471
11	Dipotassium	4.041	4.041	4.041	0.289	0.290	0.287
12	Dirubidium	3.838	3.836	3.837	0.282	0.281	0.280
13	Nitrogen	15.743	15.743	15.743	−3.227	−3.227	−3.227
14	Phosphorusdimer	10.269	10.268	10.268	0.128	0.128	0.128
15	Arsenicdimer	9.653	9.649	9.649	0.591	0.593	0.591
16	Fluorine	16.203	16.208	16.210	0.047	0.054	0.055
17	Chlorine	11.489	11.487	11.489	0.209	0.210	0.210
18	Bromine	10.663	10.672	10.679	1.008	1.009	1.009
19	Iodine	9.607	9.605	9.605	1.279	1.278	1.279
20	Methane	14.562	14.564	14.567	−2.297	−2.297	−2.297
21	Ethane	12.977	12.976	12.970	−2.267	−2.268	−2.269
22	Propane	12.317	12.321	12.283	−2.230	−2.229	−2.231
23	Buthane	11.834	11.850	11.861	−2.238	−2.237	−2.237
24	Ethylene	10.538	10.539	10.537	−2.432	−2.433	−2.430
25	Acetylene	11.295	11.295	11.299	−2.441	−2.441	−2.440
26	Tetracarbon	11.176	11.204	11.176	1.972	1.973	1.974
27	Cyclopropane	10.977	10.979	10.973	−2.423	−2.423	−2.423
28	Benzene	9.235	9.242	9.246	−1.831	−1.824	−1.819
29	Cyclooctatetraene	8.377	8.379	8.381	−0.888	−0.895	−0.890
30	Cyclopentadiene	8.612	8.624	8.604	−1.851	−1.846	−1.848
31	Vynilfluoride	10.563	10.582	10.562	−2.295	−2.296	−2.296
32	Vynilchloride	10.073	10.076	10.076	−2.157	−2.161	−2.162
33	Vynilbromide	9.225	9.267	9.266	−2.036	−2.032	−2.031
34	Vyniliodide	9.372	9.371	9.371	−1.454	−1.452	−1.452
35	Carbontetrafluoride	16.746	16.745	16.744	−2.270	−2.270	−2.270
36	Carbontetrachloride	11.664	11.665	11.665	−0.846	−0.844	−0.843
37	Carbontetrabromide	10.604	10.609	10.602	0.494	0.476	0.487
38	Carbontetraiodide	9.377	9.380	9.380	1.474	1.489	1.489
39	Silane	13.065	13.065	13.066	−1.826	−1.826	−1.826
40	Germane	12.746	12.742	12.746	−1.588	−1.590	−1.590
41	Disilane	10.865	10.864	10.865	−1.609	−1.610	−1.613
42	Pentasilane	9.478	9.473	9.479	−0.833	−0.829	−0.831
43	Lithiumhydride	8.094	8.094	8.094	0.095	0.095	0.095
44	Potassiumhydride	6.246	6.258	6.242	0.130	0.129	0.127
45	Borane	13.558	13.558	13.555	−0.622	−0.622	−0.623
46	Diborane6	12.609	12.609	12.609	−1.469	−1.469	−1.469
47	Amonia	11.032	11.033	11.042	−1.878	−1.877	−1.877
48	Hydrogenazide	10.749	10.750	10.742	−1.725	−1.725	−1.726
49	Phosphine	10.628	10.628	10.628	−1.576	−1.577	−1.576
50	Arsine	10.493	10.492	10.494	−1.542	−1.542	−1.542
51	Hydrogensulfide	10.312	10.312	10.313	−1.658	−1.658	−1.658
52	Hydrogenfluoride	16.397	16.403	16.386	−1.637	−1.636	−1.637
53	Hydrogenchloride	12.615	12.614	12.613	−1.654	−1.654	−1.654
54	Lithiumfluoride	11.745	11.774	11.748	0.168	0.167	0.167
55	Magnesiumfluoride	14.133	14.107	14.115	0.122	0.121	0.122

Continued on next page

	Name	PBE	IP PBE0	Hartree-Fock	PBE	EA PBE0	Hartree-Fock
56	Titaniumfluoride	15.729	15.732	15.743	0.582	0.581	0.578
57	Aluminumtrifluoride	15.754	15.768	15.760	−0.527	−0.526	−0.527
58	Fluoroborane	11.078	11.076	11.077	−1.664	−1.667	−1.664
59	Sulfertetrafluoride	13.121	13.125	13.122	−0.846	−0.848	−0.843
60	Potassiumbromide	8.282	8.289	8.286	0.464	0.464	0.464
61	Galliummonochloride	9.820	9.807	9.819	−0.129	−0.128	−0.131
62	Sodiumchloride	9.106	9.105	9.105	0.539	0.539	0.542
63	Magnesiumchloride	11.676	11.678	11.677	0.216	0.217	0.216
64	Aluminumtriiodide	9.875	9.882	9.884	0.270	0.274	0.275
65	Boronnitride	11.694	11.682	11.693	3.139	3.141	3.140
66	Hydrogencyanide	13.606	13.602	13.603	−2.168	−2.166	−2.167
67	Phosphorusmononitride	11.902	11.902	11.904	−0.419	−0.419	−0.419
68	Hydrazene	9.993	9.999	10.005	−1.706	−1.706	−1.705
69	Formaldehyde	11.180	11.203	11.199	−1.642	−1.637	−1.637
70	Methanol	11.417	11.426	11.423	−2.002	−2.002	−2.002
71	Ethanol	11.071	11.073	11.076	−1.972	−1.972	−1.972
72	Acetaldehyde	10.578	10.577	10.580	−1.909	−1.908	−1.908
73	Ethoxyethane	10.207	10.198	10.204	−2.183	−2.183	−2.184
74	FormicAcid	11.812	11.814	11.823	−1.955	−1.955	−1.955
75	Hydrogenperoxide	11.968	11.965	11.965	−1.813	−1.814	−1.814
76	Water	12.882	12.887	12.890	−1.638	−1.638	−1.638
77	Carbondioxide	14.014	14.011	14.012	−4.175	−4.176	−4.177
78	Carbondisulfide	9.895	9.894	9.895	−0.430	−0.432	−0.430
79	Carbonoxysulfide	11.183	11.182	11.185	−1.925	−1.927	−1.926
80	Carbonoxyselenide	10.550	10.535	10.543	−1.451	−1.453	−1.449
81	Carbonmonoxide	14.449	14.449	14.442	−1.307	−1.307	−1.307
82	Ozon	13.143	13.143	13.144	1.892	1.890	1.891
83	Sulferdioxide	12.617	12.617	12.618	0.527	0.527	0.528
84	Berylliummonoxide	10.224	10.229	10.195	1.931	1.931	1.933
85	Magnesiummonoxide	8.077	8.101	8.101	1.585	1.583	1.583
86	Tuloene	8.881	8.876	8.869	−1.754	−1.757	−1.761
87	Ethybenzene	8.781	8.781	8.801	−1.773	−1.788	−1.765
88	Hexafluorobenzene	10.183	10.199	10.196	−0.901	−0.899	−0.900
89	Phenol	8.735	8.737	8.731	−1.633	−1.630	−1.633
90	Aniline	8.030	8.039	8.031	−1.787	−1.781	−1.782
91	Pyridine	9.653	9.654	9.651	−1.237	−1.230	−1.236
92	Guanine	8.101	8.100	8.116	−1.167	−1.164	−1.144
93	Adenine	8.386	8.391	8.364	−1.233	−1.229	−1.247
94	Cytosine	8.965	8.968	8.952	−0.936	−0.935	−0.943
95	Thymine	9.277	9.278	9.281	−0.760	−0.760	−0.764
96	Uracil	9.676	9.653	9.646	−0.703	−0.706	−0.704
97	Urea	10.462	10.461	10.472	−1.481	−1.479	−1.474
98	Silverdimer	6.987	6.997	6.988	0.492	0.493	0.492
99	Copperdimer	7.611	7.610	7.607	0.351	0.351	0.352
100	Coppercyanide	10.997	10.997	10.998	0.942	0.943	0.943

2.2 TZ3P and QZ6P calculations.

For the basis set limit extrapolation, we used the qsGW@PBE0 values for TZ3P. The QZ6P values have also been calculated using PBE0 as starting point. The complete basis set limit extrapolated results have been calculated using the formula

$$\epsilon_n^{CBS} = \epsilon_n^{QZ} - \frac{1}{N_{bas}^{QZ}} \frac{\epsilon_n^{QZ} - \epsilon_n^{TZ}}{\frac{1}{N_{bas}^{QZ}} - \frac{1}{N_{bas}^{TZ}}} , \tag{S1}$$

where ϵ_n^{QZ} (ϵ_n^{TZ}) denotes the value of the quasi-particle energy using QZ6P (TZ3P) and N_{bas}^{QZ} and N_{bas}^{TZ} denote the respective numbers of basis functions. Note, that we work in spherical harmonics so that there are 5 d and 7 f functions. The number of basis functions is after canonical orthogonalization.

Table S6: Number of basis functions used for basis set limit extrapolation.

	Name	TZ3P	QZ6P
1	Helium	14	29
2	Neon	31	59
3	Argon	39	73
4	Krypton	61	111
5	Xenon	91	141
6	Hydrogen	28	53
7	Lithiumdimer	56	109
8	Sodiumdimer	72	127
9	Sodiumtetramer	144	250
10	Sodiumhexamer	216	374
11	Dipotassium	98	179
12	Dirubidium	150	256
13	Nitrogen	62	118
14	Phosphorusdimer	78	151
15	Arsenicdimer	122	217
16	Fluorine	62	119
17	Chlorine	78	152
18	Bromine	122	227
19	Iodine	182	281
20	Methane	87	157
21	Ethane	146	259
22	Propane	205	359
23	Buthane	264	462
24	Ethylene	117	209
25	Acetylene	90	157
26	Tetracarbon	124	213
27	Cyclopropane	177	310
28	Benzene	268	462
29	Cyclooctatetraene	358	615
30	Cyclopentadiene	238	411
31	Vynilfluoride	135	242
32	Vynilchloride	143	258
33	Vynilbromide	165	294
34	Vyniliodide	195	322
35	Carbontetrafluoride	155	286
36	Carbontetrachloride	187	354
37	Carbontetrabromide	275	505
38	Carbontetraiodide	395	612
39	Silane	95	176
40	Germane	118	213

Continued on next page

	Name	TZ3P	QZ6P
41	Disilane	162	300
42	Pentasilane	363	662
43	Lithiumhydride	42	83
44	Potassiumhydride	63	118
45	Borane	73	137
46	Diborane6	146	262
47	Amonia	73	136
48	Hydrogenazide	107	200
49	Phosphine	81	155
50	Arsine	103	191
51	Hydrogensulfide	67	123
52	Hydrogenfluoride	45	85
53	Hydrogenchloride	53	103
54	Lithiumfluoride	59	115
55	Magnesiumfluoride	101	180
56	Titaniumfluoride	178	329
57	Aluminumtrifluoride	132	248
58	Fluoroborane	62	112
59	Sulfertetrafluoride	163	302
60	Potassiumbromide	110	204
61	Galliummonochloride	101	187
62	Sodiumchloride	75	140
63	Magnesiumchloride	117	213
64	Aluminumtriiodide	312	492
65	Boronnitride	62	115
66	Hydrogencyanide	76	139
67	Phosphorusmononitride	70	134
68	Hydrazene	118	218
69	Formaldehyde	90	159
70	Methanol	118	208
71	Ethanol	177	310
72	Acetaldehyde	149	261
73	Ethoxyethane	295	512
74	FormicAcid	121	211
75	Hydrogenperoxide	90	160
76	Water	59	105
77	Carbondioxide	93	158
78	Carbondisulfide	109	195
79	Carbonoxysulfide	101	178
80	Carbonoxyselenide	124	215
81	Carbonmonoxide	62	110
82	Ozon	93	162
83	Sulferdioxide	101	177
84	Berylliummonoxide	62	110
85	Magnesiummonoxide	70	119
86	Tuloene	327	564
87	Ethybenzene	386	664
88	Hexafluorobenzene	371	654
89	Phenol	298	512
90	Aniline	313	542
91	Pyridine	254	443
92	Guanine	411	719
93	Adenine	380	666
94	Cytosine	318	555
95	Thymine	363	627
96	Uracil	304	526
97	Urea	180	322
98	Silverdimer	160	264
99	Copperdimer	108	189
100	Coppercyanide	116	207

Table S7: Technical parameters of the QZ6P calculations.

	Name	nIter	nBas	nTime	nFreq
1	Helium	6	29	11	11
2	Neon	13	59	17	17
3	Argon	7	73	23	23
4	Krypton	8	111	27	29
5	Xenon	6	141	27	28
6	Hydrogen	6	53	10	10
7	Lithiumdimer	27	109	14	14
8	Sodiumdimer	15	127	23	23
9	Sodiumtetramer	10	250	21	21
10	Sodiumhexamer	9	374	21	21
11	Dipotassium	8	179	26	26
12	Dirubidium	7	256	27	30
13	Nitrogen	6	118	18	18
14	Phosphorusdimer	14	151	20	20
15	Arsenicdimer	8	217	27	29
16	Fluorine	6	119	17	17
17	Chlorine	5	152	24	24
18	Bromine	6	227	27	29
19	Iodine	6	281	27	30
20	Methane	6	157	15	15
21	Ethane	6	259	15	15
22	Propane	6	359	15	15
23	Buthane	5	462	18	18
24	Ethylene	5	209	17	17
25	Acetylene	6	157	16	16
26	Tetracarbon	14	213	16	16
27	Cyclopropane	5	310	17	17
28	Benzene	5	462	16	16
29	Cyclooctatetraene	9	615	18	18
30	Cyclopentadiene	7	411	16	16
31	Vynilfluoride	7	242	18	18
32	Vynilchloride	8	258	23	23
33	Vynilbromide	6	294	27	29
34	Vyniliodide	6	322	27	32
35	Carbontetrafluoride	7	286	17	17
36	Carbontetrachloride	6	354	24	24
37	Carbontetrabromide	8	505	27	29
38	Carbontetraiodide	8	612	27	31
39	Silane	7	176	21	21
40	Germane	10	213	27	27
41	Disilane	6	300	22	22
42	Pentasilane	7	662	20	20
43	Lithiumhydride	10	83	13	13
44	Potassiumhydride	15	118	25	25
45	Borane	10	137	16	16
46	Diborane6	6	262	16	16
47	Amonia	10	136	16	16
48	Hydrogenazide	1	200	18	18
49	Phosphine	6	155	22	22
50	Arsine	10	191	27	29
51	Hydrogensulfide	6	123	23	23
52	Hydrogenfluoride	11	85	16	16
53	Hydrogenchloride	6	103	22	22
54	Lithiumfluoride	17	115	18	18
55	Magnesiumfluoride	10	180	18	18
56	Titaniumfluoride	6	329	24	24
57	Aluminumtrifluoride	6	248	21	21
58	Fluoroborane	13	112	18	18
59	Sulfertetrafluoride	6	302	23	23

Continued on next page

	Name	nIter	nBas	nTime	nFreq
60	Potassiumbromide	7	204	27	29
61	Galliummonochloride	19	187	27	29
62	Sodiumchloride	20	140	24	24
63	Magnesiumchloride	6	213	24	24
64	Aluminumtriiodide	7	492	27	32
65	Boronnitride	28	115	21	21
66	Hydrogencyanide	9	139	17	17
67	Phosphorusmononitride	14	134	22	22
68	Hydrazene	7	218	17	17
69	Formaldehyde	9	159	18	18
70	Methanol	7	208	17	17
71	Ethanol	6	310	17	17
72	Acetaldehyde	10	261	17	17
73	Ethoxyethane	6	512	17	17
74	FormicAcid	9	211	17	17
75	Hydrogenperoxide	6	160	17	17
76	Water	9	105	17	17
77	Carbondioxide	15	158	17	17
78	Carbondisulfide	11	195	23	23
79	Carbonoxysulfide	13	178	22	22
80	Carbonoxyselenide	11	215	27	29
81	Carbonmonoxide	14	110	17	17
82	Ozon	8	162	17	17
83	Sulferdioxide	7	177	23	23
84	Berylliummonoxide	21	110	18	18
85	Magnesiummonoxide	24	119	21	21
86	Tuloene	5	564	16	16
87	Ethybenzene	5	664	16	16
88	Hexafluorobenzene	6	654	18	18
89	Phenol	14	512	18	18
90	Aniline	14	542	17	17
91	Pyridine	8	443	17	17
92	Guanine	9	719	18	18
93	Adenine	6	666	17	17
94	Cytosine	11	555	18	18
95	Thymine	7	627	17	17
96	Uracil	15	526	18	18
97	Urea	9	322	18	18
98	Silverdimer	10	264	27	31
99	Copperdimer	15	189	27	29
100	Coppercyanide	20	207	26	26

Table S8: Complete Basis set limit extrapolated ionization potentials and electron affinities for the GW100 database.

	Name	IP			EA		
		TZ3P	QZ6P	extra	TZ3P	QZ6P	extra
1	Helium	24.320	24.560	24.790	−8.720	−2.770	2.780
2	Neon	21.740	21.650	21.550	−11.350	−3.780	4.600
3	Argon	15.480	15.600	15.720	−8.770	−1.910	5.950
4	Krypton	13.940	13.980	14.040	−7.420	−1.470	5.790
5	Xenon	12.270	12.290	12.320	−5.600	−1.370	6.320
6	Hydrogen	16.490	16.480	16.460	−3.070	−2.420	−1.700
7	Lithiumdimer	5.260	5.340	5.420	0.120	0.180	0.250
8	Sodiumdimer	5.000	5.040	5.100	0.220	0.270	0.330
9	Sodiumtetramer	4.250	4.340	4.450	0.470	0.520	0.580
10	Sodiumhexamer	4.390	4.450	4.530	0.470	0.480	0.490
11	Dipotassium	4.040	4.120	4.230	0.290	0.340	0.410
12	Dirubidium	3.840	3.910	4.010	0.280	0.330	0.400
13	Nitrogen	15.740	15.860	15.980	−3.230	−2.260	−1.180
14	Phosphorusdimer	10.270	10.370	10.480	0.130	0.360	0.600
15	Arsenicdimer	9.650	9.650	9.660	0.590	0.630	0.670
16	Fluorine	16.210	16.260	16.310	0.050	0.050	0.050
17	Chlorine	11.490	11.600	11.720	0.210	0.430	0.660
18	Bromine	10.670	10.720	10.770	1.010	1.050	1.090
19	Iodine	9.600	9.630	9.680	1.280	1.360	1.510
20	Methane	14.560	14.620	14.690	−2.300	−1.620	−0.780
21	Ethane	12.980	13.020	13.080	−2.270	−1.560	−0.650
22	Propane	12.320	12.350	12.390	−2.230	−1.510	−0.560
23	Buthane	11.850	11.900	11.970	−2.240	−1.500	−0.520
24	Ethylene	10.540	10.650	10.790	−2.430	−1.670	−0.700
25	Acetylene	11.290	11.420	11.590	−2.440	−1.960	−1.320
26	Tetracarbon	11.200	11.390	11.640	1.970	2.220	2.570
27	Cyclopropane	10.980	11.070	11.180	−2.420	−1.650	−0.610
28	Benzene	9.240	9.360	9.520	−1.820	−1.350	−0.690
29	Cyclooctatetraene	8.380	8.490	8.640	−0.900	−0.710	−0.460
30	Cyclopentadiene	8.620	8.730	8.880	−1.850	−1.500	−1.040
31	Vynilfluoride	10.580	10.660	10.770	−2.300	−1.620	−0.760
32	Vynilchloride	10.080	10.160	10.270	−2.160	−1.490	−0.650
33	Vynilbromide	9.270	9.370	9.490	−2.030	−1.570	−0.980
34	Vyniliodide	9.370	9.430	9.520	−1.450	−1.120	−0.620
35	Carbontetrafluoride	16.750	16.760	16.780	−2.270	−2.920	−3.690
36	Carbontetrachloride	11.660	11.730	11.800	−0.840	−0.570	−0.270
37	Carbontetrabromide	10.610	10.640	10.690	0.480	0.520	0.570
38	Carbontetraiodide	9.380	9.410	9.460	1.490	1.580	1.760
39	Silane	13.060	13.100	13.140	−1.830	−1.460	−1.040
40	Germane	12.740	12.760	12.790	−1.590	−1.450	−1.280
41	Disilane	10.860	10.890	10.930	−1.610	−0.930	−0.130
42	Pentasilane	9.470	9.520	9.570	−0.830	0.670	2.500
43	Lithiumhydride	8.090	8.160	8.230	0.100	0.120	0.150
44	Potassiumhydride	6.260	6.380	6.530	0.130	0.190	0.250
45	Borane	13.560	13.580	13.610	−0.620	−0.430	−0.200
46	Diborane6	12.610	12.640	12.690	−1.470	−1.310	−1.100
47	Amonia	11.030	11.080	11.130	−1.880	−1.310	−0.650
48	Hydrogenazide	10.750	10.870	11.010	−1.720	−1.230	−0.670
49	Phosphine	10.630	10.670	10.710	−1.580	−1.380	−1.170
50	Arsine	10.490	10.590	10.700	−1.540	−1.280	−0.980
51	Hydrogensulfide	10.310	10.410	10.520	−1.660	−1.110	−0.460
52	Hydrogenfluoride	16.400	16.380	16.360	−1.640	−1.360	−1.060
53	Hydrogenchloride	12.610	12.710	12.820	−1.650	−1.180	−0.670
54	Lithiumfluoride	11.770	11.790	11.810	0.170	0.220	0.280
55	Magnesiumfluoride	14.110	14.080	14.040	0.120	0.170	0.220
56	Titaniumfluoride	15.730	15.820	15.920	0.580	0.670	0.770

Continued on next page

	IP			EA			
	Name	TZ3P	QZ6P	extra	TZ3P	QZ6P	extra
57	Aluminumtrifluoride	15.770	15.750	15.720	−0.530	−0.260	0.050
58	Fluoroborane	11.080	11.130	11.200	−1.670	−1.290	−0.830
59	Sulfertetrafluoride	13.130	13.120	13.120	−0.850	−0.630	−0.380
60	Potassiumbromide	8.290	8.310	8.330	0.460	0.490	0.510
61	Galliummonochloride	9.810	9.940	10.100	−0.130	−0.050	0.050
62	Sodiumchloride	9.110	9.220	9.360	0.540	0.590	0.660
63	Magnesiumchloride	11.680	11.800	11.940	0.220	0.300	0.410
64	Aluminumtriiodide	9.880	9.900	9.920	0.270	0.360	0.510
65	Boronnitride	11.680	11.820	11.980	3.140	3.330	3.550
66	Hydrogencyanide	13.600	13.710	13.850	−2.170	−1.470	−0.620
67	Phosphorusmononitride	11.900	11.990	12.100	−0.420	−0.130	0.190
68	Hydrazene	10.000	10.040	10.090	−1.710	−1.150	−0.500
69	Formaldehyde	11.200	11.230	11.260	−1.640	−1.450	−1.210
70	Methanol	11.430	11.440	11.460	−2.000	−1.460	−0.750
71	Ethanol	11.070	11.110	11.160	−1.970	−1.400	−0.640
72	Acetaldehyde	10.580	10.630	10.710	−1.910	−1.450	−0.850
73	Ethoxyethane	10.200	10.270	10.360	−2.180	−1.510	−0.600
74	FormicAcid	11.810	11.870	11.950	−1.960	−1.530	−0.960
75	Hydrogenperoxide	11.970	11.980	12.010	−1.810	−1.340	−0.730
76	Water	12.890	12.880	12.870	−1.640	−1.290	−0.850
77	Carbondioxide	14.010	14.080	14.180	−4.180	−1.410	2.550
78	Carbondisulfide	9.890	10.040	10.230	−0.430	−0.160	0.180
79	Carbonoxysulfide	11.180	11.330	11.520	−1.930	−1.080	0.020
80	Carbonoxyselenide	10.540	10.600	10.690	−1.450	−1.280	−1.040
81	Carbonmonoxide	14.450	14.520	14.620	−1.310	−1.040	−0.710
82	Ozon	13.140	13.250	13.400	1.890	2.020	2.190
83	Sulferdioxide	12.620	12.670	12.730	0.530	0.690	0.910
84	Berylliummonoxide	10.230	10.220	10.200	1.930	2.060	2.220
85	Magnesiummonoxide	8.100	8.140	8.200	1.580	1.720	1.910
86	Tuloene	8.880	8.990	9.140	−1.760	−1.370	−0.840
87	Ethybenzene	8.780	8.950	9.180	−1.790	−1.310	−0.640
88	Hexafluorobenzene	10.200	10.270	10.360	−0.900	−0.810	−0.680
89	Phenol	8.740	8.840	8.990	−1.630	−1.320	−0.890
90	Aniline	8.040	8.170	8.350	−1.780	−1.210	−0.420
91	Pyridine	9.650	9.760	9.910	−1.230	−1.010	−0.720
92	Guanine	8.100	8.200	8.330	−1.160	−0.750	−0.190
93	Adenine	8.390	8.460	8.550	−1.230	−1.060	−0.830
94	Cytosine	8.970	9.050	9.170	−0.930	−0.750	−0.500
95	Thymine	9.280	9.340	9.420	−0.760	−0.600	−0.370
96	Uracil	9.650	9.740	9.870	−0.710	−0.550	−0.330
97	Urea	10.460	10.520	10.600	−1.480	−0.970	−0.330
98	Silverdimer	7.000	7.060	7.150	0.490	0.570	0.690
99	Copperdimer	7.610	7.660	7.730	0.350	0.440	0.560
100	Coppercyanide	11.000	11.070	11.160	0.940	1.100	1.300

2.3 Results using augmented basis sets

Table S9: Complete Basis set limit extrapolated ionization potentials and electron affinities for the GW100 database.

IP				EA			
	Name	TZ3P	QZ6P	extra	TZ3P	QZ6P	extra
Methane	14.58	14.610	14.660	−0.790	−0.580	−0.260	
Ethane	13.00	13.020	13.070	−0.720	−0.570	−0.350	
Propane	12.33	12.350	12.380	−0.720	−0.550	−0.300	
Buthane	11.86	11.890	11.950	−0.710	−0.550	−0.300	
Ethylene	10.56	10.630	10.730	−0.850	−0.610	−0.220	
Acetylene	11.36	11.430	11.530	−0.990	−0.630	−0.050	
Vynilfluoride	10.65	10.670	10.690	−0.570	−0.570	−0.560	
Vynilchloride	10.09	10.180	10.330	−0.760	−0.420	0.090	
Vynilbromide	9.27	9.340	9.440	−0.760	−0.540	−0.220	
Carbontetrafluoride	16.68	16.760	16.890	−0.500	−0.840	−1.350	
Carbontetrachloride	11.66	11.750	11.890	−0.530	−0.240	0.170	
Silane	13.00	13.110	13.280	−0.650	−0.550	−0.390	
Germane	12.75	12.770	12.790	−0.530	−0.480	−0.410	
Disilane	10.83	10.900	11.000	−0.510	−0.500	−0.480	
Borane	13.54	13.580	13.640	−0.340	−0.270	−0.180	
Diborane6	12.59	12.630	12.680	−0.710	−0.540	−0.300	
Amonia	11.03	11.080	11.160	−0.660	−0.460	−0.180	
Hydrogenazide	10.78	10.850	10.930	−0.580	−0.390	−0.120	
Phosphine	10.65	10.680	10.740	−0.570	−0.490	−0.370	
Arsine	10.49	10.580	10.710	−0.560	−0.460	−0.310	
Hydrogensulfide	10.37	10.400	10.460	−0.570	−0.280	0.150	
Hydrogenfluoride	16.37	16.320	16.260	−0.450	−0.430	−0.410	
Hydrogenchloride	12.63	12.730	12.870	−0.570	−0.300	0.050	
Fluoroborane	11.08	11.160	11.300	−0.540	−0.660	−0.870	
Hydrogencyanide	13.63	13.720	13.860	−0.700	−0.430	−0.030	
Formaldehyde	11.18	11.190	11.210	−0.700	−0.490	−0.130	
Methanol	11.39	11.450	11.540	−0.680	−0.500	−0.220	
Ethanol	11.07	11.110	11.160	−0.650	−0.490	−0.240	
Acetaldehyde	10.58	10.630	10.710	−0.660	−0.450	−0.130	
Ethoxyethane	10.21	10.260	10.340	−0.700	−0.520	−0.230	
Water	12.85	12.900	12.970	−0.560	−0.410	−0.190	
Urea	10.47	10.510	10.570	−0.460	−0.270	0.010	

REFERENCES
